# Antimicrobial-Resistant *Listeria monocytogenes* in Ready-to-Eat Foods: Implications for Food Safety and Risk Assessment

**DOI:** 10.3390/foods12061346

**Published:** 2023-03-22

**Authors:** Adeoye John Kayode, Anthony Ifeanyi Okoh

**Affiliations:** 1 Applied and Environmental Microbiology Research Group (AEMREG), Department of Biochemistry and Microbiology, University of Fort Hare, Private Bag X1314, Alice 5700, South Africa; 2 SAMRC Microbial Water Quality Monitoring Center, University of Fort Hare, Private Bag X1314, Alice 5700, South Africa; 3 Department of Environmental Health Sciences, College of Medical and Health Sciences, University of Sharjah, Sharjah P.O. Box 27272, United Arab Emirates

**Keywords:** antibiotic resistance, empirical treatment, prescribed antibiotics, listeriosis, antibiotics

## Abstract

Antimicrobial resistance is an existential threat to the health sector, with far-reaching consequences in managing microbial infections. In this study, one hundred and ninety-four *Listeria monocytogenes* isolates were profiled for susceptibility using disc diffusion techniques. Possible foodborne listeriosis risk associated with ready-to-eat (RTE) foods (RTEF) and the risk of empirical treatment (EMPT) of *L. monocytogenes* infections, using multiple antimicrobial resistance indices (MARI) and antimicrobial resistance indices (ARI), respectively, were investigated. Twelve European Committee on Antimicrobial Susceptibility Testing (EUCAST) prescribed/recommended antimicrobials (EPAS) for the treatment of listeriosis and ten non-prescribed antimicrobials (non-PAS)] were evaluated. Antimicrobial resistance > 50% against PAs including sulfamethoxazole (61.86%), trimethoprim (56.19%), amoxicillin (42.27%), penicillin (41.24%), and erythromycin (40.21%) was observed. Resistance > 50% against non-PAS, including oxytetracycline (60.89%), cefotetan (59.28%), ceftriaxone (53.09%), and streptomycin (40.21%) was also observed. About 55.67% and 65.46% of the isolates had MARI scores ranging from 0.25–0.92 and 0.30–0.70 for EPAs and non-PAs, respectively. There was a significant difference (*p* < 0.01) between the MARI scores of the isolates for EPAs and non-PAs (means of 0.27 ± 0.21 and 0.31 ± 0.14, respectively). MARI/ARI scores above the Krumperman permissible threshold (>0.2) suggested a high risk/level of antimicrobial-resistant *L. monocytogenes.* The MARI risks of the non-success of empirical treatment (EMPT) attributed to EPAs and non-PAs were generally high (55.67% and 65.463%, respectively) due to the antimicrobial resistance of the isolates. MARI-based estimated success and non-success of EMPT if EUCAST-prescribed antimicrobials were administered for the treatment of listeriosis were 44.329% and 55.67%, respectively. The EMPT if non-prescribed antimicrobials were administered for the treatment of listeriosis was 34.53% and 65.46%, respectively. This indicates a potentially high risk with PAs and non-PAs for the treatment of *L. monocytogenes* infection. Furthermore, ARI scores ≤ 0.2 for EPAs were observed in polony, potato chips, muffins, and assorted sandwiches, whereas ARI scores for non-PAs were >0.2 across all the RTE food types. The ARI-based estimate identified potential risks associated with some RTE foods, including fried fish, red Vienna sausage, Russian sausage, fruit salad, bread, meat pies, fried chicken, cupcakes, and vetkoek. This investigation identified a high risk of EMPT due to the presence of antimicrobial-resistant *L. monocytogenes* in RTE foods, which could result in severe health consequences.

## 1. Introduction

Antimicrobials are substances that kill or inhibit microbial growth and could be synthetic, semi-synthetic, or derived originally from natural sources. They are used specifically to prevent or treat bacterial infections. Antimicrobial discovery is a breakthrough in modern medicine. However, this success might be obliterated by the exponential increase of antimicrobial resistance among pathogenic microorganisms with a far-reaching impact on human health [1,2,3]. Antimicrobial resistance impairs the capacity of the human immune system to fight infections. It contributes to complications in at-risk patients having chronic health conditions like arthritis, asthma, rheumatoid, and diabetes. Patients undergoing chemotherapy, dialysis, joint replacement, and surgery are also vulnerable. Furthermore, the emergence of new superbugs, treatment failures, high mortality, and morbidity rates are major effects of antimicrobial resistance. It has also been established that antimicrobial-resistant pathogens will increase the probability of the occurrence of a serious health issue twofold and triple the chances of death compared to the non-resistant ones [4,5] if not accorded adequate attention.

Apart from the health impact highlighted earlier, antimicrobial resistance has serious economic consequences. For instance, the financial implication of antimicrobial resistance is extremely high due to an increase in drug usage and hospital admissions, and this differs in each country. The Centre for Disease Control (CDC) estimated a yearly cost of 20 billion USD in the United States for healthcare and 35 billion USD in productivity loss [4,5]. In 2015, Alessandro and colleagues estimated the cases of infection with some antimicrobial-resistant bacteria (AMB) in the EU to be 671,689 [6]. The estimated burden directly associated with drug-resistant infections globally was 1.27 million deaths in 2019 [7]. Furthermore, de Kraker et al. estimated that death due to the challenge of antimicrobial resistance will exceed 10 million and a cumulative cost of 1 trillion USD to global productivity per year by 2050 if the global response to antibiotic resistance is not put on global alert [8,9]. Hence, the WHO has declared antimicrobial resistance a global problem [10]. In the food sector, antimicrobial resistance has potentially led to greater food safety concerns, a reduction in food production, economic losses to farm households, and reduced food security.

Antimicrobial misuse has a significant impact on resistance selection in bacteria and the emergence of resistance is most pronounced within the hospital environmental ecological niches and the community. In a survey of 155 students, Davey et al. observed that a reduction in excessive antimicrobial prescriptions is associated with a decline in *Clostridium difficile* colonization and infections or infection with cephalosporin, aminoglycoside-resistant Gram-negative bacteria, vancomycin-resistant *Enterococcus faecalis*, and methicillin-resistant *Staphylococcus aureus* (MRSA). Interventions targeted at increasing effective prescription also improved clinical outcomes [11,12]; as such, there is a close connection between antimicrobial misuse/abuse and resistance development. The excessive use of antimicrobials in aquaculture, food animal production, and crop culture has contributed immensely to the challenge of antimicrobial resistance. Antimicrobials are administered not only for prophylactics and metaphylaxis but also as growth promoters to boost food animal production for the teaming human population [1,13].

In the food chain, antimicrobial-resistant bacteria represent a potential global public health threat. This is because the ecosystem of the food production chain is ecologically composed of various niches, where there is a co-existence of numerous bacteria, and many antimicrobials are used. Vegetables, food animals, and fishes are known to be reservoirs of antimicrobial-resistant bacteria. Along the food chain, humans encounter resistant bacteria when contaminated foods or food products (e.g., meat, eggs, dairy products) are consumed or through direct contact with infected animals or biological fluids from animals.

There have been reports describing the presence of large numbers of AMB and ARGs in various foods like bulk milk, ready-to-eat foods, and cooked meats at different stages of production [1,14,15,16]. Reports of clonally related AMB (including *L. monocytogenes*) and ARGs from foods have also been identified in human populations with no history of occupational exposures. These provide evidence of transmission of AMB due to food consumption or handling [17,18]. Given the health consequence associated with AMB in foods, our present study evaluated the antimicrobial susceptibility of *L. monocytogenes* from RTE foods. Based on the result obtained, the associated risk (listeriosis) with RTE foods (RTEF) and the risk of empirical treatment (EMPT) of *L. monocytogenes* infections when prescribed and non-prescribed antimicrobials are used for the management of listeriosis were evaluated. This was achieved using the data generated from multiple antimicrobial resistance indices (MARI) and antimicrobial resistance indices (ARI), respectively. This report provided a background to the antibiotic sensitivity of *Listeria monocytogenes* from RTE foods in the Eastern Cape Province, South Africa (ECPSA), as an indicator for the evaluation of RTEF and EMPT.

## 2. Methodology

### 2.1. Study Area

This study was carried out to estimate the possible risks associated with antimicrobial-resistant *L. monocytogenes* from RTE foods and the risk of empirical treatment at three Municipality Districts (Sarah Baartman, Chris Hani, and Amathole) of the Eastern Cape Province, South Africa. These municipalities occupy 33.57° S, 25.36° E, 31.8743° S, 26.7968° E, and 32.5842° S, 27.3616° E of the geographical coordinates on the map, respectively.

#### 2.1.1. Sample Collection

Two hundred and thirty-nine (239) ready-to-eat food samples of thirteen different food types popularly consumed by many South Africans were randomly selected at the sampling points. The food samples included polony (soft-textured sausage made of beef and pork enclosed in a hued red or orange skin), Russian sausage or kolbasa (made from ground meat—poultry or pork, beef, along with spices, flavorings, and salt) wrapped in a special casing, fruit salad (blends of fruits in a sweet sauce made from juice and honey), potato chips, Vienna sausage (soft, meaty, red-skinned, fine textured sausage produced from mechanically deboned chicken, pork, spices, salt, vegetable protein and other ingredients), fried fish, vetkoek/fat cake/amagwinya (South African fried dough that is fluffy inside and crispy outside and often stuffed with savory or sweet fillings), meat pie, bread, fried chicken, assorted sandwiches (made from lemon rind, cream cheese, bread slices, lettuce, sausages, and tomato) muffins and cupcakes were obtained in supermarkets/grocery stores at different points in towns and cities within the municipalities chosen for this study. The samples were collected between February and September 2019. Samples were collected aseptically from the sampling points and wrapped in labeled sterile plastic bags to avoid cross-contamination. They were within 6 h conveyed in iced insulated boxes to the laboratory for analysis.

#### 2.1.2. Enumeration of Presumptive Listeria in RTE Food Samples

Presumptive aerobic plate count was carried out according to the standard methods of the International Organization for Standardization (EN ISO 11290-2:2017. Twenty-five grams of each sample was aseptically stomached in 225 mL of Buffered Peptone Water (BPW, Oxoid Ltd., Basingstoke, Hampshire, UK). The samples were serially diluted in three replicates of tenfold dilutions and 0.5 mL of the dilutions were plated on *Listeria* Chromogenic agar, as previously described [19].

### 2.2. Detection of L. monocytogenes

*L. monocytogenes* detection in ready-to-eat food samples was undertaken employing the guidelines of EN ISO 11290-1:2017. A 25 g of each aseptically stomached sample was pre-enriched and plated on selective media using the procedure previously described [19]. Blue colonies surrounded by halos were subcultured on Tryptone Soy Agar to purify the isolates. The purified cultures of the presumptive isolates were preserved at –80 °C in Tryptone Soy Broth with 25% glycerol.

#### 2.2.1. DNA Extraction

Genomic DNA isolation was carried out by the boiling method before the molecular confirmation of the isolates described previously [20,21]. Presumptive isolates previously preserved in 25% glycerol stock were grown in 5 mL Tryptone Soy Broth (CM0129 Oxoid Ltd., Basingstoke, Hampshire, UK) at 37 °C for 18 h to resuscitate the isolates. The broths were transferred into 2 mL Eppendorf tubes and centrifuged at 16,000 rpm for 5 min using a mini-spin micro-centrifuge. The supernatants were removed after centrifugation, the pellets were washed in normal saline, and 300 μL nuclease-free water was added to the pellet and allowed to boil in a heating block (TECHNE Digital Dri-Block DB-3D, London, UK) at 100 °C for 10 min. After boiling, the samples were removed from the heating block and left to cool for 10 min on ice. The samples were centrifuged at 16,000 rpm for 5 min to remove the cell debris. The DNA template (supernatant) was transferred into a clean Eppendorf tube and kept at 20 °C for further analysis.

#### 2.2.2. Molecular Characterization of *L. monocytogenes* Isolates

Presumptive isolates were screened for the *Listeria* genus using the 370 base pairs (370 bp) section of the 16S rRNA *prs* gene. Amplification of this segment was made using the primer sets F-GCTGAAGAGATTGCGAAAGAAG and R-CAAAGAAACCTTGGATTTGCGG as described previously [22]. Polymerase chain reaction (PCR) was carried out in a thermal cycler (BIO-RAD T100) using the cycling condition 94 °C: 5 min; 33 cycles (94 °C: 45 s; 56 °C: 30 s, 72 °C: 1 min, 72 °C: 5 min). The *iap* (invasion-associated protein) gene targeting *L. monocytogenes* at 131 base pair (bp) using primer set: F-ACAAGCTGCACCTGTTGCAG and R-TGACAGCGTGTGTAGTAGCA was amplified by PCR using the above-mentioned thermal cycler. All PCR reactions were prepared in a final volume (25 µL) containing 12.5 µL master mix (One taq Quick Load 2 × Master mix; BioLabs Inc., Hitchin, UK), 1 µL of *prs*/*iap* primers, 0.5 µL buffer, and MgCl_2_, and 6.5 µL of sterile nuclease-free water. The PCR cycling condition [94 °C: 5 min; 35 cycles (94 °C: 35 s; 52 °C: 30 s, 72 °C: 1 min; 72 °C: 10 min)] was optimized using positive controls to validate the procedure. The gel electrophoresis system (ADVANCE Mupid™-One, Takara, Japan) was used to separate the PCR products in agarose and detected 131 bp with Alliance 4.7 UV trans-illuminator (Alliance XD-79.WL/26MX, Paris, France). Referenced strains of *L. monocytogenes* (ATCC 19118 and ATCC 7644) were used as positive controls, and nuclease-free water was used as the negative control. The PCR products were sequenced using a Sanger sequencer to further verify the isolates’ identity. Some of the sequences (OL694843, OL694844, OL694845) were submitted to NCBI GenBank.

### 2.3. Antimicrobial Susceptibility Testing (AST)

Testing for the antimicrobial susceptibility of *L. monocytogenes* isolates adopted the Kirby Bauer disc diffusion method in conformity with the standard procedure described by the European Committee on Antimicrobial Susceptibility Testing (EUCAST) [23]. The test organisms were tested for susceptibility against 22 different antimicrobials (Table S1a) belonging to the β-lactams, aminoglycosides, carbapenems, cephalosporin, glycopeptides, macrolides, fluoroquinolones, sulfonamides, tetracyclines, phenicol, phosphonic acid derivative, and colistin sulphate. The antimicrobials were classified into two distinct groups [12 EUCAST-prescribed antimicrobials (EPAs) for the treatment of listeriosis infections and 10 first-line antimicrobials for the empirical treatment of infections caused by pathogenic microorganisms (non-prescribed antibiotics for treatment of microbial infections)]. The referenced strains of *L. monocytogenes* (ATCC 7644 and ATCC 19118) served as the positive control. The isolates’ susceptibility was categorized as susceptible (S), resistant (R) or intermediate (I) to each of the antimicrobials, in line with the result obtained from the susceptibility testing using standard reference documents (Table S1a) [23].

#### 2.3.1. Computation of Resistance Quotient (RQs) of *L. monocytogenes* Isolates

The frequency of antimicrobial resistance phenotypes of the RTE food isolates was calculated for each antimicrobial across all the RTE food samples [24].

(1) $$\mathrm{Resistant}\;\mathrm{quotient}=\frac{\mathrm{No}.\;\mathrm{of}\;\mathrm{antimicrobial}\;\mathrm{resistant}\;\mathrm{isolates}\;\mathrm{from}\;a\;\mathrm{particular}\;\mathrm{food}\;\mathrm{sample}\;}{\mathrm{Total}\;\mathrm{no}.\;\mathrm{of}\;\mathrm{isolates}\;\mathrm{from}\;\mathrm{the}\;\mathrm{sample}}\times 100\;$$

#### 2.3.2. Antimicrobial Resistance Phenotyping, Multiple Antimicrobial Resistance Indexing of Isolates and Risk Evaluation

Twelve EUCAST-prescribed antimicrobials (EPAs) for the treatment of listeriosis infections and ten first-line antimicrobials for the empirical treatment of microbial infections (non-prescribed antibiotics for treatment of listeriosis, non-PAs) were investigated for Multiple Antimicrobial Resistance Indices (MARI) and Antimicrobial Resistance Index (ARI) scores. The Multiple Antimicrobial Resistance Phenotypes (MARPs) of *L. monocytogenes* in respect of EPAs and non-PAs were computed for each of the isolates that showed resistance against three or more antimicrobials and indexed for MARI values [25]. The MARI scores were calculated as follows:

(2) $$\mathrm{MARI}\;\mathrm{index}=\frac{\mathrm{no}.\;\mathrm{of}\;\mathrm{antibiotics}\;\mathrm{to}\;\mathrm{which}\;\mathrm{isolate}\;\mathrm{was}\;\mathrm{resistant}}{\mathrm{no}.\;\mathrm{of}\;\mathrm{antibiotics}\;\mathrm{to}\;\mathrm{which}\;\mathrm{isolate}\;\mathrm{was}\;\mathrm{exposed}}$$

The MARI of isolates above the Krumperman threshold (>0.2) indicated exposure of the isolates to high antimicrobial selection pressure in the region.

In addition, the Antimicrobial Resistance Index (ARI) was computed for each of the RTE food samples as described by [25]. Thus, ARI for PAs and non-PAs was computed.

(3) $$\mathrm{ARI}=\frac{a}{b}$$

where *a* = aggregate antibiotic resistance score of all isolates from a sample; *b* = number of antimicrobial resistance score of all isolates from a sample

ARI > 0.2 suggested a high level of antimicrobial-resistant *L. monocytogenes* associated with a particular RTE food.

The frequency of resistance, the number of antimicrobials against which the isolates are resistant, and the pattern of multiple antimicrobial resistance were described.

The possible risks associated with RTE food (RTEF) and the risk of empirical treatment (EMPT) were investigated by comparing the MARI and ARI scores of the EPAs and non-Pas, respectively, using the statistical model described in a previous report [24]. The risks were computed and interpreted based on the outlined assumptions construed around the arbitrary Krumperman value [25]:

The MARI (risk) with EPA_S_ for treatment of *L. monocytogenes* infection is often lower (MARI_EPAs_ ≤ 0.2, when PAs are used for treatment) as against when non-PAs are used for the treatment of infections (MARI_non-PAs_ > 0.2). The arbitrary MARI or ARI threshold (0.2) was adopted to identify/differentiate high and low risks [25].

When isolates are susceptible to EPAs for therapy, EMPT = 0; if otherwise, EMPT > 0. The EMPT of listeriosis infection is illustrated thus: MARI_EMPT_ = MARI_EPAs_ + MARI_non-PAs_ (Where either of MARI_EPAs_ + MARI_non-PAs_ = 0, depending on the selected group of antimicrobials for empirical treatment).

The ARI for each RTE food sample is ≤0.2 when EPAs are assessed, only if there is no antimicrobial resistance selection pressure (ARI_EPAs_ ≤ 0.2), whereas ARI_non-PAs_ > 0.2 (when non-PAs are assessed) in any event of the presence or absence of antimicrobial resistance selection pressure. Consequently, the RTEF of a particular RTE food based on the profiling of antimicrobial resistance is defined as ARI_RTEF_ = ARI_EPAs_ + ARI_non-PAs_ (where either ARI_EPAs_ + ARI_non-PAs_ = 0, depending on the group selected for AST).

### 2.4. Data Analysis

The data obtained were processed for descriptive analysis. The resistance quotients (RQs) across all the RTE foods were computed using Microsoft Excel Sheet, Microsoft Corporation 365, Bellevue, WA, USA. (Retrieved from https://office.microsoft.com/excel, accessed on 28 October 2021). Hierarchical clustering of the antibiotic susceptibility test results was achieved by K-mean and visualized by “ComplexHeatmap” OriginPro 2023 (version 10.0) statistical software, Northampton, MA, USA. The differences between MARI and ARI of the two groups of antimicrobials were subjected to Wilcoxon signed-rank test for comparison. Values were considered statistically significant at *p* < 0.01 and *p* < 0.05.

## 3. Results

### 3.1. Occurrence of L. monocytogenes in RTE Foods

**Figure 1:**
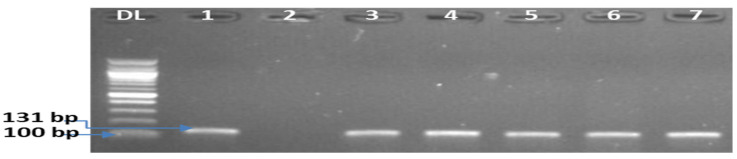
A representation of the electrophoresis gel image of the PCR products by the simplex PCR showing the gene fragment (131 bp) for the confirmation of *L. monocytogenes*. Lane DL: 100 bp DNA ladder, lane 1: +ve control, lane 2: −ve control (*Listeria monocytogenes* ATCC 19118), lane 3–7 positive *L. monocytogenes* isolates.

The presumptive counts observed from the lowest to the highest ranged between 1.0 × 10^3^–2.7 × 10^6^ CFU/g. Higher presumptive counts were recorded from meat pie, fried fish, sliced polony, cupcakes, Russian sausages, bread, and potato chips. This indicates a higher risk of *L. monocytogenes* in these foods compared with other ones in this study. RTE foods that had 0 CFU/g and less than 10 CFU/g presumptive counts were more in number compared with those with 10–100 and >100 CFU/g (Table 1). One hundred and ninety-four *L. monocytogenes* isolates were detected in the ready-to-eat food samples as follows: 20 from polony; 23 from sliced polony; 30 from fruit salad; 16 from chips; 21 from fried fish; 4 from Vienna sausages; 14 from Russian sausages; 11 from bread; 2 from fried chicken; 22 from meat pie; 10 from cupcakes; 12 from muffins, and 9 from assorted sandwiches Table 1. Figure 1 shows the gel electrophoresis image of confirmed *L. monocytogenes.*

### 3.2. Antibiotic Susceptibility and Cluster Analysis of L. monocytogenes Isolates

*L. monocytogenes* (194) isolates were profiled for susceptibility to 22 antimicrobials (12 EPA_S_ and 10 non-PA_S_). Figures S1–S3 described the phenotypic antimicrobial pattern of susceptibility of each of the *L. monocytogenes* isolates to both EPA_S_ and non-PA_S_. This pattern observed reflects the resistance attributes as it revealed the efficacy of the antimicrobials towards each isolate. Susceptibility to EPAs (>50%) was observed and ranged from 57.73 (amoxicillin) to 94.85 (ampicillin-sulbactam), except for erythromycin (27.32) and sulfamethoxazole (34.02%), that had low susceptibility rates. Susceptibility > 50% to non-PA_S_ ranged from 59.28% (vancomycin) to 81.96% (fosfomycin) except for cefotetan, ceftriaxone, and oxytetracycline with 31.44, 29.90, and 29.38%, respectively. However, resistance > 50% against EPAs, including sulfamethoxazole (61.86%), trimethoprim (56.19%), amoxicillin (42.27%), penicillin (41.24%), and erythromycin (40.21%) were observed. Furthermore, resistance > 50% against non-PA_S,_ including oxytetracycline (60.89%), cefotetan (59.28%), ceftriaxone (53.09%), and streptomycin (40.21%) were observed (Table S1b).

#### 3.2.1. Prevalence of Antimicrobial-Resistant *L. monocytogenes* and Computation of Resistance Quotient (RQs) of Isolates

The distribution of antimicrobial-resistant *L. monocytogenes* across RTE food samples is provided in Table 2. Phenotypic resistance of *L. monocytogenes* against various antibiotics in each RTE food sample ranged between 1 to 22. Table 2 describes the RQs of the isolates to various antimicrobials ranging from 3.33 to 100%. Higher RQs of antimicrobial against EPAs, including penicillin, amoxicillin, ertapenem, trimethoprim, sulfamethoxazole, and non-PAs, including streptomycin, ceftriaxone, cefotetan, oxytetracycline were observed in RTE foods such as red Vienna, cupcake, Russian sausage, fruit salad, bread, fried fish, fried chicken, potato chips, pies, and muffins. Lower RQs were recorded for EPAs, including ampicillin, ampicillin–sulbactam, doripenem, imipenem, clarithromycin, and trimethoprim–sulfamethoxazole. Lower RQs were also observed for non-PAs including gentamicin, amikacin, ciprofloxacin, chloramphenicol, and fosfomycin across the RTE food samples. A significant (*p* < 0.01) relationship in the distribution of phenotypically resistant *L. monocytogenes* isolates and all RTE foods was observed.

#### 3.2.2. Multiple Antimicrobial Resistance Phenotypes and Index (MARPs and MARI) of *L. monocytogenes*

The MARPs patterns and MARI are provided in Table 3. Ready-to-eat food *L. monocytogenes* displayed 65 patterns of MARPs for EPAs ranging from 3 to 11 antimicrobials, while 63 MARPs patterns were observed for non-PAs ranging from 3 to 7 antimicrobials. The P/AML/W/RL (*n* = 12) MARP occurred most for PAs, while the MARPs that occurred once were the most predominant. The CRO/CCT/OT phenotype (*n* = 21) occurred most among the MAR phenotypes observed. For non-PAs, the MARPs that occurred once were the most predominant. Twenty-seven (*n* = 27, 13.92%) of the isolates were not resistant against any of the EPAs, 34 (17.53%) showed resistance against one of the EPAs, 24 (12.37%) showed resistance against at least 2 of the EPAs. In comparison, 108 (55.67%) exhibited multiple resistance phenotypes against EPAs. Also, 24 (12.37%) of the isolates were resistant against one non-PAs, 41 (21.13%) showed resistance against two non-PAs, and 127 (65.46%) showed multiple antibiotic resistance against non-PAs. The phenotypic resistance patterns against both EPAs and non-PAs and the prevalence of *L. monocytogenes* in each RTE food are provided in Table 3.

### 3.3. Evaluation of the RTEF and the EMPT Entrenched on the MAR and ARI of L. monocytogenes Isolates

#### EMPT from the Comparison of MARI of *L. monocytogenes*

The comparative assessment of MARI of EPAs and non-PAs antimicrobials is presented in Table 3. The difference between the MARI scores of the isolates for EPAs and non-PAs with a means of 0.27 ± 0.21 (median = 0.33, mode = 0.80) and 0.31 ± 0.14 (mean = 0.30, mode = 0.30), respectively, were significant (*p* < 0.01). Table S2 revealed that 86 (44.329%) and 108 (55.67%) of *L. monocytogenes* isolates had MARI of 0–0.17 and 0.25–0.92 for EPAs, respectively. In addition, 67 (34.53%) and 127 (65.463%) of the isolates had MARI ranging between 0–0.2 and 0.3–70 for non-Pas, respectively. The MARI of the RTE food isolates for EPAs ranged between 0.25 and 0.92 for EPAs and non-PAs, ranging between 0.30 and 0.70, and are greater than the permissible (0.2) benchmark. This indicates high-risk contamination of the RTE foods. Notably, the responses of the isolates to EPAs and non-PAs varied considerably. While some isolates had zero MARI scores for EPAs, they had MARI > 0.2 for non-PAs.

In summary, the success of EMPT (MARI ≤ 0.2) and non-success of EMPT (MARI > 0.2) of *L. monocytogenes* infections due to RTE food *L. monocytogenes* isolates for EPAs represent 44.329 and 55.67%, respectively. In like manner, the success of EMPT (MARI ≤ 0.2) and non-success of EMPT (MARI > 0.2) of *L. monocytogenes* infections due to RTE food isolates for non-PAs represent 34.53 and 65.463%, respectively. In any case, risk varied with individual antibiotics and isolates.

### 3.4. RTEF from the Comparison of MARI of L. monocytogenes Isolates

#### RTEF from the Comparison of ARI across the Ready-to-Eat Foods

**Figure 2:**
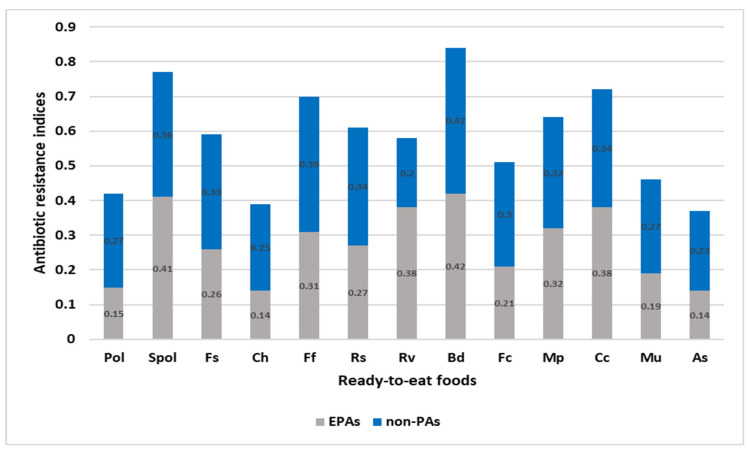
The comparative antimicrobial resistance index (ARI) scores across all ready-to-eat foods tested were represented. ARI scores across non-PAs > 0.2 across all the RTE foods except red Vienna (ARI = 0.2). ARI < 0.2 for PAs for polony, potato chips, muffins, and assorted sandwiches. Spol—sliced polony, Pol—polony, Fs—fruit salad, Ff—fried fish, Ch—chips, Rs—Russian sausage, Bd—bread, Rv—red Vienna, Fc—fried chicken, Cc—cupcakes, Mp—meat pie, As—assorted sandwiches, Mu—muffins.

The comparative ARI scores across all ready-to-eat foods tested are provided in Figure 2. The differences in ARI across the ready-to-eat foods for EPAs (ARI average 3.58) and non-PAs (ARI average 4.02) were not significant (*p* > 0.01). The ARI scores ≤ 0.2 for EPAs were observed in polony, potato chips, muffins, and assorted sandwiches, whereas ARI scores for non-PAs were >0.2 across all the RTE food types except red Vienna (ARI = 0.2). The ARI-based estimation identified potential risks associated with some RTE foods such as Russian sausage, fruit salad, fried fish, red Vienna sausage, bread, meat pies, vetkoek, fried chicken, and cupcakes.

## 4. Discussion

This study evaluated the antimicrobial susceptibility of *L. monocytogenes* against antimicrobial agents currently in use for managing listeriosis and the potential possible risks of antimicrobial resistance. Useful information/insight from the data obtained could guide relevant authorities in decision-making and preparedness to mitigate public health emergencies. The detection of *L. monocytogenes* in the RTE food samples we analyzed could suggest unhygienic practices/exposure to contamination from humans/surfaces of processing facilities and cross-contamination after processing; this is also attributable to the resilience of *L. monocytogenes* contributing to the high prevalence observed in RTE foods.

Foodborne infection (listeriosis) caused by *L. monocytogenes* is often acquired when foods contaminated with the vegetative cells of the pathogen are consumed. It is one of the major infections affecting food safety, causing human illness worldwide with significant public health and economic impact [26]. In this study, the aerobic plate count revealed that <100 CFU/g was observed in 96.77% of the food samples processed. This conforms with the 100 CFU/g permissible limit of *L. monocytogenes* in foods in the EU. However, 55.23% met the zero-tolerance adopted in the USA, whereas 44.77% of the foods failed the zero-tolerance permissible limit. The actual infective dose of *L. monocytogenes* widely accepted is not yet documented. Nonetheless, a previous European Food Safety Authority study stated that over 90% of listeriosis is attributable to ingesting foods with more than 2000 CFU/g, and 33% is attributable to the proliferation of *L. monocytogenes* in foods at the storage or consumption phase [27]. Foods including meat pie, fried fish, sliced polony, cupcakes, Russian sausages, bread, and potato chips had higher counts compared with others. This indicates a higher risk of *L. monocytogenes* in the RTE foods considering that *L. monocytogenes* has the capacity to proliferate in foods in storage, even at refrigeration temperatures. The detection of *L. monocytogenes* in RTE foods in our study agrees with the reports from previous studies on processed foods, including fish products, meats, and delicatessens in Poland [28]; pate, cheese, shellfish, and sausages in Chile [29].

According to the WHO, foodborne infection attributed to *L. monocytogenes* is caused by multi-drug resistant strains [30]. This is because antimicrobial-resistant pathogens have different strategies they employ to defeat the efficacy of antimicrobial drugs, including reduced protein synthesis, resistance to the inhibition of nucleotide synthesis, cell membrane disruption, transport-based mechanisms by protecting the ribosomal binding site of tetracycline via RNA binding proteins and fluoroquinolones resistance due to topoisomerase IV genes and DNA gyrase mutations [30,31]. Several reports revealing the emergence of antimicrobial-resistant foodborne pathogens, especially *L. monocytogenes* in the food chain, have been published [32,33,34,35]. The existence of antimicrobial-resistant bacteria in food may lead to difficulty treating foodborne infections in humans. Their presence in food can also facilitate the transfer of resistant genes to other microorganisms through the food chain [36,37,38]. In our study, the phenotypic resistance observed against streptomycin, sulfamethoxazole, trimethoprim, cefotetan, oxytetracyclines vancomycin, and ceftriaxone was similar to previous reports on antibiotic resistance of *L. monocytogenes* from food tested against several antimicrobials [34,35,39,40]. The high level of resistance observed against these antimicrobials could suggest a gradual decline in their efficacy for the treatment of listeriosis [39] due to drug misuse or the residual impact of antimicrobials in the environment. This could also be attributed to the resistance acquired during adaptation to environmental stresses like heat, desiccation, and biological stress due to microbial antagonism that could induce cross-protection responses, giving rise to cells with increased resistance. Furthermore, the development of resistance to stress, such as oxidants, irradiation, and elevated pressure in food production processes, could also occur [41,42]. A previous study reported that exposure to cold, salt stress, and pH increased *L. monocytogenes* resistance against different antimicrobials [43]. Notably, the high resistance observed against certain non-prescribed antimicrobials (non-PAs) could describe the intrinsic resistance of *L. monocytogenes* against such antimicrobials. The RQs values recorded for certain antimicrobials across the RTE food matrixes could likely indicate the pressure of antimicrobial selection in the region.

The comparative assessment of MARI of EUCAST prescribed antimicrobial and non-prescribed antimicrobial suggests the underlying risk which may be involved in the treatment of listeriosis cases with the antimicrobial groups. In essence, the estimated success of empirical treatment (EMPT) of listeriosis infection in our study indicated that when treatment of foodborne listeriosis is required in this study area, clinicians that select the prescribed antimicrobials for EMPT, after diagnosis was properly made have a 44.329% chance of preventing fatal treatment outcomes and 55.67% non-success compared with 34.53% successful and 65.463% non-successful chance of preventing fatal outcomes for selecting non-PAs for empirical treatment. Although, treatment of listeriosis is challenging, primarily because the highest percent of affected patients is usually immunocompromised due to comorbidity or immune impairment related to aging or weak immunity as in the case of infants. In this regard, empirical treatment using a drug active against *L. monocytogenes* was advised because they are usually found to be associated with reduced mortality [44]. Late or incorrect diagnoses and wrongly prescribed antimicrobials for treatment could also lead to a high mortality rate [44,45]. There have been non-consistent reports of success and non-success of empirical treatment of listeriosis infection. Bateman et al. reported the success of empirical treatment of listeriosis patients treated with amoxicillin, ceftriaxone, ampicillin, acyclovir, rifampicin, isoniazid, dexamethasone, methylprednisolone, trimethoprim/sulfamethoxazole, and metronidazole [46]. However, reports of non-success of empirical treatment involving antimicrobials, including telithromycin, moxifloxacin, methylprednisolone, acyclovir, ceftriaxone, dexamethasone, ampicillin, gentamycin, chloramphenicol, and trimethoprim/sulfamethoxazole were documented [47,48]. Furthermore, a combination of azithromycin and ceftriaxone, imipenem, and ampicillin, cotrimoxazole, and ampicillin for empirical treatment of listeriosis infection was unsuccessful in South Africa [47]. Some of the antibiotics employed in treatment here were not prescribed by the CLSI/EUCAST [23,49]. However, an ideal active antimicrobial against *L. monocytogenes* must be able to penetrate the host cell and bind to intracellular target tightly” [50,51,52]. Some scholars attempted to investigate the suitability of certain antimicrobials for alleviating listeriosis infection, but they all arrived at divergent opinions. Among active antimicrobials against *L. monocytogenes*, ampicillin, amoxicillin, and penicillin are the most commonly used and supported by expert guidelines/opinions [53]. Some scholars also put forward that a synergistic combination of aminopenicillin (ampicillin and amoxicillin) and gentamicin as the reference treatment of *L. monocytogenes* infection would be appropriate to alleviate listeriosis [51,54]. A previous randomized study described by de Gans found that dexamethasone did not worsen outcomes when administered in patients with non-pneumococcal meningitis [50]. However, another study revealed a harmful effect of dexamethasone adjunct in neurolisteriosis in a subset of patients and advocated the use of cotrimoxazole, gentamicin, and beta-lactam over other antimicrobials [45]. Furthermore, the strength and limitations of the therapeutic use of cotrimoxazole [45,55,56], quinolones [57], levofloxacin [58,59], linezolid [60,61], meropenem [62,63,64], rifampin [65], vancomycin [66] were documented. The challenge of non-consistent outcomes as regards successes and non-successes of antimicrobials used for empirical treatment could be a result of a lack of adequate clinical trials and non-existing evidence-based medical management of listeriosis cases [45]. Although, this scenario is also dependent on the high rate of resistance acquisition/ARGs among *L. monocytogenes*, which largely influence the efficacy of antibiotics against the pathogen.

ARI observed was not a good indicator for the evaluation of EMPT for both EPAs and non-PAs, as the group means were >0.2. ARI for EPAs was <0.2 in RTE foods including potato chips, polony, assorted sandwiches, and muffins, while ARI for non-PAs (ARI = 0.2) was observed for red Vienna. Higher ARI in other RTE foods could be an indication of antimicrobial selection pressures and high-risk contamination of foods within the study area.

Of note, the approach of comparing MARI/ARI between EPAs and non-PAs against *L. monocytogenes* calls for caution when the Krumperman’s threshold (0.2) is applied [25] to establish low/high-risk contamination of *L. monocytogenes* in RTE foods. Most importantly, the MAR/AR Indices of intrinsically resistant microbes against certain antimicrobials are usually ≥0.2. Most isolates that have MARI < 0.1 to EPAs in this study were observed with a value ≥ 0.2 for non-PAs (Table S4).

Summarily, this study revealed the antimicrobial resistance profiles and the potential health risks due to antimicrobial resistance. High resistance (>50%) against amoxicillin, penicillin, ertapenem, erythromycin, sulfamethoxazole, cefotetan, ceftriaxone, trimethoprim, streptomycin, oxytetracyclines, and vancomycin was observed. The resistance against antimicrobials among *L. monocytogenes* indicates the possible health risk that could arise from the consumption of such foods, especially among immunocompromised persons. MARI evaluation disclosed a high risk of EMPT of listeriosis in EPAs and non-PAs. The chance of successful EMPT is generally low but a little higher for PAs. ARI based on EPAs revealed potential risk across all RTE foods except polony, potato chips, muffins, and assorted sandwiches, while the AR Index for non-PAs in all RTE foods was above the Krumperman value except for red Vienna alone. We, therefore, suggest a more intensified campaign against antimicrobial misuse and prioritizing the search for novel antimicrobial agents that can serve as an alternative option for the treatment of listeriosis. Lastly, newly updated clinical trials for evidence-based medical management could improve the success of empirical treatments of listeriosis, to address the challenge of non-consistent outcomes regarding successes and non-successes of antimicrobials for the empirical treatment of foodborne listeriosis are required.

## Figures and Tables

**Table 1:** Distribution of *L. monocytogenes* in ready-to-eat foods and presumptive aerobic plate count.

Type of Samples	Samples Tested	Presumptive Counts of *Listeria* in RTE Food Samples (cfu/g)				RTE Foods Positive for *L. monocytogenes* (%)	*L. monocytogenes* in RTE Foods (%)
		>100	10–100	<10	0		
Fruit salad	20	0	3	7	10	10/20 (50)	30 (15.46)
Fried fish (snoek)	21	2	3	3	13	8 (38.10)	21 (10.82)
Sliced polony	21	3	5	5	8	13 (61.90)	23 (11.85)
Polony	19	0	1	9	9	10 (52.63)	20 (10.30)
Russian sausage	14	2	3	6	3	11 (78.57)	14 (7.21)
Bread	21	0	3	3	15	6 (28.57)	11 (5.67)
Chips	21	0	1	9	11	10 (47.62)	16 (8.24
Vetkoek	21	0	0	0	21	0	0
Cupcakes	21	1	1	7	12	9 (42.86)	10 (5.15)
Vienna sausages	8	0	1	3	4	4 (50)	4 (2.06)
Meat pie	21	0	2	9	10	11 (52.38)	22 (11.34)
Fried chicken	5	0	1	1	3	2 (40)	2 (1.03)
Assorted sandwiches	14	0	1	5	8	6 (42.86)	9 (4.63)
Muffins	12	1	0	6	5	7 (58.33)	12 (6.18)
Total (%)	239	9 (3.77)	25 (10.46)	73 (30.54)	132 (55.23)	107 (44.77)	194 (100)

**Table 2:** Antibiotic RQs (%) of *L. monocytogenes* in RTE food samples.

RTE Food	N	P	AMP	SAM	AML	CN	AK	S	DOR	ETP	IPM	CRO	CTT	VA	E	CLA	CIP	W	RL	TS	OT	C	FOS
Pol	20	30.00	0.00	0.00	30.00	5.00	10.00	30.00	15.00	5.00	35.00	45.00	65.00	30.00	30.00	5.00	5.00	50.00	50.00	5.00	60.00	5.00	15.00
SPol	23	60.87	39.13	30.44	47.82	17.39	8.70	52.17	4.35	60.87	21.74	47.83	60.87	60.87	47.83	26.09	13.04	65.22	69.57	34.78	65.22	26.09	8.70
FS	30	30.00	0.00	0.00	50.00	13.33	23.33	33.33	30.00	36.67	6.67	70.00	3.33	36.67	36.67	6.67	3.33	46.67	73.33	0.00	66.67	23.33	3.33
Ch	16	12.50	6.25	6.25	18.75	6.25	6.25	18.75	6.25	31.25	18.75	31.25	93.75	12.50	18.75	12.50	0.00	25.00	18.75	6.25	62.50	12.50	6.25
FF	21	57.14	0.00	0.00	33.33	9.52	38.1	47.62	23.81	33.33	19.05	76.19	95.23	47.62	47.62	19.05	0.00	66.67	52.38	19.05	61.91	9.52	0.00
RS	14	42.86	7.14	7.14	71.43	7.14	0.00	50.00	7.14	21.43	0.00	35.71	50.00	14.29	35.71	7.14	14.29	50.00	78.57	28.57	92.86	14.29	21.43
RV	4	75.00	25.00	25.00	100	0.00	0.00	25.00	0.00	75.00	0.00	0.00	0.00	100	0.00	0.00	0.00	75.00	75.00	25.00	75.00	0.00	0.00
Bd	11	54.55	0.00	0.00	54.55	27.27	36.36	72.72	36.36	27.27	0.00	72.72	72.27	81.82	90.91	27.27	0.00	100	90.91	18.18	45.46	9.09	0.00
FC	2	0.00	0.00	0.00	50.00	0.00	50.00	0.00	0.00	50.00	0.00	100	100	0.00	50.00	0.00	50.00	50.00	50.00	0.00	0.00	0.00	0.00
Ps	22	40.91	4.55	0.00	50.00	4.55	36.36	45.45	9.09	50.00	18.18	63.64	81.82	40.91	50.00	22.73	9.09	59.09	59.09	27.27	72.73	13.64	0.00
Cc	10	70.00	0.00	0.00	40.00	0.00	10.00	60.00	40.00	50.00	10.00	50.00	70.00	50.00	50.00	10.00	10.00	90.00	80.00	10.00	70.00	10.00	10.00
Mu	12	33.33	0.00	0.00	25.00	0.00	8.33	25.00	16.67	8.33	0.00	25.00	83.33	25.00	16.67	16.67	0.00	41.67	66.67	8.33	66.67	0.00	25.00
AS	9	22.22	0.00	0.00	11.11	11.11	0.00	22.22	0.00	66.67	0.00	44.44	0.00	0.00	33.33	0.00	0.00	33.33	44.44	0.00	44.44	0.00	11.11

Pol—polony, Spol—sliced polony, FS—fruit salad, Ch—potato chips, FF—fried fish, RS—Russian sausage, RV—red Vienna, Bd—bread, FC—fried chicken, Vk—vetkoek, Ps—pie, Cc—cupcakes, Mu—muffins, AS—assorted sandwiches. Antimicrobials: penicillin G (P), ampicillin (AMP), ampicillin–sulbactam (SAM), amoxicillin (AML), gentamicin (CN), amikacin (AK), streptomycin (S), doripenem (DOR), ertapenem (ETP), imipenem (IPM), ceftriaxone (CRO), cefotetan (CTT), vancomycin (VA), erythromycin (E), clarithromycin CLA, ciprofloxacin (CIP), trimethoprim (W), sulfamethoxazole (RL), trimethoprim–sulfamethoxazole (TS), oxytetacyclin (OT), chloramphenicol (C), fosfomycin (FOS). Regions highlighted in green represents RQs > 50% while red represents RQs ≥ 70%.

**Table 3:** Comparison of the multiple antibiotic resistance phenotypes of *L. monocytogenes* to EUCAST prescribed and non-prescribed antibiotics.

	MARPs (Prescribed Antibiotics)	No of Antibiotics	No Observed	MARI	MARPs (Non-Prescribed)	No of Antibiotics	No Observed	MARI
1	AML/E/CLA	3	1	0.25	AK/S/OT’	3	1	0.30
2	AML/ETP/RL	3	1	0.25	AK/OT/C	3	1	0.30
3	AMP/ETP/TS	3	1	0.25	AK/CRO/CTT	3	2	0.30
4	AML/IPM/RL	3	3	0.25	AK/CRO/CCT/OT	4	7	0.40
5	AML/W/RL	3	2	0.25	AK/S/CRO/CCT	4	2	0.40
6	AML/IPM/W	3	1	0.25	AK/S/CRO/CTT/VA	5	5	0.50
7	AML/DOR/RL	3	1	0.25	AK/S/CRO/CCT/OT	5	4	0.50
8	AML/RL/TS	3	1	0.25	AK/CRO/CCT/VA/OT	5	3	0.50
9	AML/DOR/IPM	3	1	0.25	AK/S/CRO/CCT/OT	5	1	0.50
10	AML/E/W/RL	4	2	0.33	AK/CRO/CCT/OT/C	5	1	0.50
11	AML/ETP/W/RL	4	2	0.33	AK/CRO/CCT/VA/OT/C	6	1	0.60
12	AML/IPM/E/W/RL	5	2	0.41	AK/S/CRO/CCT/VA/OT	6	2	0.60
13	AML/ETP/E/W/RL	5	2	0.41	CIP/OT/C	3	1	0.30
14	AML/E/W/RL/TS	5	1	0.41	CN/S/VA	3	1	0.30
15	AML/DOR/CLA/W/RL/TS	6	2	0.50	CCT/OT/C	3	2	0.30
16	AML/DOR/E/CLA/W/RL/TS	7	1	0.58	CN/S/CRO	3	1	0.30
17	AMP/SAM/AML/IPM/W/RL/TS	7	1	0.58	CRO/CTT/CIP	3	1	0.30
18	AMP/SAM/AML/DOR/CLA/W/RL/TS	8	1	0.67	CRO/CIP/OT	3	1	0.30
19	DOR/E/W/RL	4	1	0.33	CRO/CCT/C	3	1	0.30
20	DOR/IPM/ETP	3	1	0.25	CCT/VA/OT	3	3	0.30
21	DOR/ETP/E/CLA/W/RL/TS	7	1	0.58	CRO/CCT/VA	3	1	0.30
22	E/W/RL	3	4	0.25	CRO/CCT/OT	3	21	0.30
23	E/CLA/W	3	1	0.25	CCT/VA/FOS	3	2	0.30
24	E/W/RL/TS	4	1	0.33	CRO/VA/OT/C	4	5	0.40
25	E/CLA/W/RL/TS	5	1	0.42	CRO/CCT/VA/OT	4	3	0.40
26	IPM/E/W	3	2	0.25	CN/S/CCT/OT	4	1	0.40
27	IPM/ETP/E	3	1	0.25	CN/S/CRO/VA	4	1	0.40
28	IPM/E/W/RL	4	1	0.33	CRO/CCT/OT/C	4	2	0.40
29	IPM/ETP/E/RL	4	1	0.33	CN/CCT/VA/OT	4	1	0.40
30	IPM/W/RL/IPM	4	1	0.33	CCT/VA/CIP/OT	4	1	0.40
31	IPM/ETP/W/RL	4	1	0.33	CRO/CCT/CIP/OT	4	1	0.40
32	P/W/RL	3	1	0.25	CN/VA/OT/FOS	4	1	0.40
33	P/E/W/RL	4	3	0.33	CN/CCT/OT/FOS	4	1	0.40
34	P/AML/W/RL	4	12	0.33	CN/S/CCT/VA/OT	5	1	0.50
35	P/AML/E/RL	4	1	0.33	CN/S/CCT/OT/FOS	5	1	0.50
36	P/IPM/W/RL	4	1	0.33	CRO/CCT/OT/FOS	4	1	0.40
37	P/AML/IPM/W	4	3	0.33	CRO/VA/CIP/OT/C	5	2	0.50
38	P/AML/E/W/RL	5	4	0.42	CN/CRO/CTT/VA/OT	5	1	0.50
39	P/DOR/E/W/RL	5	1	0.42	CN/S/CRO/CTT/VA	5	2	0.50
40	P/IPM/E/W/RL	5	1	0.42	CN/AKS/VA/OT/FOS	5	1	0.50
41	P/AML/IMP/W/RL	5	3	0.42	CN/AK/S/CRO/CCT/OT	6	1	0.60
42	P/AML/DOR/W/RL	5	1	0.42	CN/AK/S/CRO/CCT/VA	6	1	0.60
43	P/AML/DOR/E/W/RL	6	2	0.50	CN/AK/S/CRO/CCT/OT/C	7	1	0.70
44	P/AML/E/CLA/W/RL	6	2	0.50	CN/CRO/CCT/VA/CIP/OT/C	7	1	0.70
45	P/AML/IPM/E/W/RL	6	4	0.50	S/VA/OT	3	4	0.30
46	P/IPM/ETP/E/W/RL	6	1	0.50	S/OT/FOS	3	2	0.30
47	P/DOR/IPM/E/W/RL	6	1	0.50	S/CCT/VA	3	3	0.30
48	P/AML/CLA/W/RL/TS	6	2	0.50	S/CCT/OT	3	2	0.30
49	P/AML/DOR/IPM/W/RL	6	1	0.50	S/CCT/FOS	3	1	0.30
50	P/AML/IPM/ETP/E/W	6	1	0.50	S/CRO/CCT	3	1	0.30
51	P/AML/E/CLA/W/RL/TS	7	1	0.58	S/VA/OT/C	4	1	0.40
52	P/AML/DOR/IPM/E/W/RL	7	2	0.58	S/CRO/VA/OT	4	2	0.40
53	P/AML/IPM/ETP/E/W/RL	7	1	0.58	S/CRO/CCT/OT	4	2	0.40
54	P/AML/DOR/CLA/W/RL/TS	7	5	0.58	S/CRO/CCT/VA	4	3	0.40
55	P/AMP/SAM/DOR/E/RL/TS	7	1	0.58	S/CRO/VA/OT/C	5	1	0.50
56	P/AML/DOR/E/CLA/W/RL/TS	8	1	0.67	S/CCT/VA/OT/C	5	1	0.50
57	P/AML/DOR/IPM/E/CLA/W/RL	8	1	0.67	S/VA/CIP/OT/C	5	1	0.50
58	P/AMP/SAM/IPM/E/CLA/W/RL/TS	9	2	0.75	S/CRO/CCT/OT/C	5	1	0.50
59	P/AMP/SAM/AML/E/CLA/W/RL/TS	9	1	0.75	S/CRO/CCT/VA/OT	5	3	0.50
60	P/AML/DOR/IPM/ETP/E/CLA/W/RL	9	1	0.75	S/CRO/CCT/VA/OT/C	6	1	0.60
61	P/AMP/SAM/AML/DOR/CLA/W/RL/TS	9	1	0.75	S/CCT/VA/CIP/OT/C	6	1	0.60
62	P/AMP/SAM/AML/IPM/E/CLA/W/RL/TS	10	1	0.83	S-CRO-VA-OT-FOS	5	1	0.50
63	P/AMP/SAM/AML/IPM/E/CLA/W/RL/TS	10	1	0.83	VA/OT/C	3	1	0.30
64	P/AMP/SAM/AML/IPM/ETP/E/CLA/W/RL/TS	11	1	0.92				

## Data Availability

Data is contained within the article or Supplementary Material.

## Supplementary Materials

The following supporting information can be downloaded at: https://www.mdpi.com/article/10.3390/foods12061346/s1, Figure S1: Heatmap cluster analysis of *L. monocytogenes* isolates from ready-to-eat foods. The Column and row clusters grouped isolates and antimicrobials according to the response/susceptibility. Figure S2. Heatmap cluster analysis of *L. monocytogenes* isolates from ready-to-eat foods. The Column and row clusters grouped isolates and antimicrobials according to the response/susceptibility. Figure S3. Heatmap cluster analysis of *L. monocytogenes* isolates from ready-to-eat foods. The Column and row clusters grouped isolates and antimicrobials according to the response/susceptibility. Table S1a: Antibiotic breakpoints for the description of the antibiotic susceptibility testing for *L. monocytogenes.* Table S1b: Description of antibiotic susceptibility profile of *L. monocytogenes* isolates (n = 194) recovered from RTE food. Table S2: Multiple/Antibiotic resistance index of *L. monocytogenes* to EUCAST recommended and non-recommended antibiotics.

## References

[1] Founou L.L., Founou R.C., Essack S.Y. (2016). Antibiotic resistance in the food chain: A developing country-perspective. Front. Microbiol..

[2] Hashempour-Baltork F., Hosseini H., Shojaee-Aliabadi S., Torbati M., Alizadeh A.M., Alizadeh M. (2019). Drug resistance and the prevention strategies in food borne bacteria: An update review. Adv. Pharm. Bull..

[3] Ilievska N., Pavlova V., Ilievska J., Kirovska V., Pavlovska M. (2019). Review paper on the effects of antibiotic use in agricultural animals on the human health and formation of food born antibiotic resistant microorganisms. J. Hyg. Eng. Des..

[4] Prestinaci F., Pezzotti P., Pantosti A. (2015). Antimicrobial resistance: A global multifaceted phenomenon. Pathog. Glob. Health.

[5] Frieden T. (2013). Antibiotic Resistance Threats in the United States, 2013|Antibiotic/Antimicrobial Resistance Report.

[6] Cassini A., Högberg L.D., Plachouras D., Quattrocchi A., Hoxha A., Simonsen G.S., Colomb-Cotinat M., Kretzschmar M.E., Devleesschauwer B., Cecchini M. (2019). Attributable deaths and disability-adjusted life-years caused by infections with antibiotic-resistant bacteria in the EU and the European Economic Area in 2015: A population-level modelling analysis. Lancet Infect. Dis..

[7] Murray C.J., Ikuta K.S., Sharara F., Swetschinski L., Robles Aguilar G., Gray A., Han C., Bisignano C., Rao P., Wool E. (2022). Global burden of bacterial antimicrobial resistance in 2019: A systematic analysis. Lancet.

[8] De Kraker M.E.A., Stewardson A.J., Harbarth S. (2016). Will 10 Million People Die a Year due to Antimicrobial Resistance by 2050?. PLoS Med..

[9] World Bank By 2050, Drug-Resistant Infections Could Cause Global Economic Damage on par with 2008 Financial Crisis. Press Release 2016. https://www.worldbank.org/en/news/press-release/2016/09/18/by-2050-drug-resistant-infections-could-cause-global-economic-damage-on-par-with-2008-financial-crisis.

[10] Bloom G., Merrett G.B., Wilkinson A., Lin V., Paulin S. (2017). Antimicrobial resistance and universal health coverage. BMJ Glob. Health.

[11] Davey P., Marwick C.A., Scott C.L., Charani E., Mcneil K., Brown E., Gould I.M., Ramsay C.R., Michie S. Interventions to improve antibiotic prescribing practices for hospital inpatients. Cochrane Database Syst. Rev..

[12] Davey P., Brown E., Charani E., Fenelon L., Gould I.M., Holmes A., Ramsay C.R., Wiffen P.J., Wilcox M. Interventions to improve antibiotic prescribing practices for hospital inpatients. Cochrane Database Syst. Rev..

[13] Newell D.G., Koopmans M., Verhoef L., Duizer E., Aidara-Kane A., Sprong H., Opsteegh M., Langelaar M., Threfall J., Scheutz F. (2010). Food-borne diseases—The challenges of 20 years ago still persist while new ones continue to emerge. Int. J. Food Microbiol..

[14] Liu Y.Y., Wang Y., Walsh T.R., Yi L.X., Zhang R., Spencer J., Doi Y., Tian G., Dong B., Huang X. (2016). Emergence of plasmid-mediated colistin resistance mechanism MCR-1 in animals and human beings in China: A microbiological and molecular biological study. Lancet Infect. Dis..

[15] Coetzee J., Corcoran C., Prentice E., Moodley M., Mendelson M., Poirel L., Nordmann P., Brink A.J. (2016). Emergence of plasmid-mediated colistin resistance (MCR-1) among *Escherichia coli* isolated from South African patients. South African Med. J..

[16] Kayode A.J., Okoh A.I. (2022). Antibiotic resistance profile of *Listeria monocytogenes* recovered from ready-to-eat foods surveyed in South Africa. J. Food Prot..

[17] Jansen W., Müller A., Grabowski N.T., Kehrenberg C., Muylkens B., Al Dahouk S. (2019). Foodborne diseases do not respect borders: Zoonotic pathogens and antimicrobial resistant bacteria in food products of animal origin illegally imported into the European Union. Vet. J..

[18] Samtiya M., Matthews K.R., Dhewa T., Puniya A.K. (2022). Antimicrobial Resistance in the Food Chain: Trends, Mechanisms, Pathways, and Possible Regulation Strategies. Foods.

[19] Kayode A.J., Okoh A.I. (2022). Assessment of the molecular epidemiology and genetic multiplicity of *Listeria monocytogenes* recovered from ready-to-eat foods following the South African listeriosis outbreak. Sci. Rep..

[20] Kayode A.J., Semerjian L., Osaili T., Olapade O., Okoh A.I. (2021). Occurrence of Multidrug-Resistant *Listeria monocytogenes* in Environmental Waters: A Menace of Environmental and Public Health Concern. Front. Environ. Sci..

[21] Kayode A.J., Okoh A.I. (2022). Incidence and genetic diversity of multi-drug resistant *Listeria monocytogenes* isolates recovered from fruits and vegetables in the Eastern Cape Province, South Africa. Int. J. Food Microbiol..

[22] Doumith M., Buchrieser C., Glaser P., Jacquet C., Martin P. (2004). Differentiation of the major *Listeria monocytogenes* serovars by multiplex PCR. J. Clin. Microbiol..

[23] EUCAST (2020). The European Committee on Antimicrobial Susceptibility Testing. Breakpoint Tables for Interpretation of MICs and Zone Diameters. Version 10.0.

[24] Ekundayo T.C., Okoh A.I. (2020). Antimicrobial resistance in freshwater Plesiomonas shigelloides isolates: Implications for environmental pollution and risk assessment. Environ. Pollut..

[25] Krumperman P.H. (1983). Multiple antibiotic resistance indexing of *Escherichia coli* to identify high-risk sources of fecal contamination of foods. Appl. Environ. Microbiol..

[26] Abebe E., Gugsa G., Ahmed M. (2020). Review on Major Food-Borne Zoonotic Bacterial Pathogens. J. Trop. Med..

[27] Ricci A., Allende A., Bolton D., Chemaly M., Davies R., Fernández Escámez P.S., Girones R., Herman L., Koutsoumanis K., Nørrung B. (2018). *Listeria monocytogenes* contamination of ready-to-eat foods and the risk for human health in the EU. EFSA J..

[28] Szymczak B., Szymczak M., Trafiałek J. (2020). Prevalence of *Listeria* species and *L. monocytogenes* in ready-to-eat foods in the West Pomeranian region of Poland: Correlations between the contamination level, serogroups, ingredients, and producers. Food Microbiol..

[29] Montero D., Bodero M., Riveros G., Lapierre L., Gaggero A., Vidal R.M., Vidal M. (2015). Molecular epidemiology and genetic diversity of *Listeria monocytogenes* isolates from a wide variety of ready-to-eat foods and their relationship to clinical strains from listeriosis outbreaks in Chile. Front. Microbiol..

[30] Bhattacharjee R., Nandi A., Mitra P., Saha K., Patel P., Jha E., Panda P.K., Singh S.K., Dutt A., Mishra Y.K. (2022). Theragnostic application of nanoparticle and CRISPR against food-borne multi-drug resistant pathogens. Mater. Today Bio.

[31] Kunjachan S., Rychlik B., Storm G., Kiessling F., Lammers T. (2013). Multidrug resistance: Physiological principles and nanomedical solutions. Adv. Drug Deliv. Rev..

[32] Mpundu P., Mbewe A.R., Muma J.B., Mwasinga W., Mukumbuta N., Munyeme M. (2021). A global perspective of antibiotic-resistant *Listeria monocytogenes* prevalence in assorted ready to eat foods: A systematic review. Vet. World.

[33] Abdeen E.E., Mousa W.S., Harb O.H., Fath-Elbab G.A., Nooruzzaman M., Gaber A., Alsanie W.F., Abdeen A. (2021). Prevalence, antibiogram and genetic characterization of *Lsteria monocytogenes* from food products in Egypt. Foods.

[34] Li L., Olsen R.H., Ye L., Wang W., Shi L., Yan H., Meng H. (2016). Characterization of Antimicrobial Resistance of *Listeria monocytogenes* Strains Isolated from a Pork Processing Plant and Its Respective Meat Markets in Southern China. Foodborne Pathog. Dis..

[35] Conter M., Paludi D., Zanardi E., Ghidini S., Vergara A., Ianieri A. (2009). Characterization of antimicrobial resistance of foodborne *Listeria monocytogenes*. Int. J. Food Microbiol..

[36] Khatibi S.A., Hamidi S., Siahi-Shadbad M.R. (2021). Current trends in sample preparation by solid-phase extraction techniques for the determination of antibiotic residues in foodstuffs: A review. Crit. Rev. Food Sci. Nutr..

[37] González-Gutiérrez M., García-Fernández C., Alonso-Calleja C., Capita R. (2020). Microbial load and antibiotic resistance in raw beef preparations from northwest Spain. Food Sci. Nutr..

[38] Rajaei M., Moosavy M.H., Gharajalar S.N., Khatibi S.A. (2021). Antibiotic resistance in the pathogenic foodborne bacteria isolated from raw kebab and hamburger: Phenotypic and genotypic study. BMC Microbiol..

[39] Du X.J., Zhang X., Wang X.Y., Su Y.L., Li P., Wang S. (2017). Isolation and characterization of *Listeria monocytogenes* in Chinese food obtained from the central area of China. Food Control.

[40] Doménech E., Jimenez -Belenguer A., Amoros J.A., Ferrus M.A., Escriche I. (2015). Prevalence and antimicrobial resistance of *Listeria monocytogenes* and *Salmonella* strains isolated in ready-to-eat foods in Eastern Spain. Food Control.

[41] Olaimat A.N., Al-Holy M.A., Shahbaz H.M., Al-Nabulsi A.A., Abu Ghoush M.H., Osaili T.M., Ayyash M.M., Holley R.A. (2018). Emergence of Antibiotic Resistance in *Listeria monocytogenes* Isolated from Food Products: A Comprehensive Review. Compr. Rev. Food Sci. Food Saf..

[42] Blair J.M.A., Webber M.A., Baylay A.J., Ogbolu D.O., Piddock L.J.V. (2015). Molecular mechanisms of antibiotic resistance. Nat. Rev. Microbiol..

[43] Aarestrup F.M., Knöchel S., Hasman H. (2007). Antimicrobial susceptibility of *Listeria monocytogenes* from food products. Foodborne Pathog. Dis..

[44] Pagliano P., Arslan F., Ascione T. (2017). Epidemiology and treatment of the commonest form of listeriosis: Meningitis and bacteraemia. Infez. Med..

[45] Charlier C., Perrodeau É., Leclercq A., Cazenave B., Pilmis B., Henry B., Lopes A., Maury M.M., Moura A., Goffinet F. (2017). Clinical features and prognostic factors of listeriosis: The MONALISA national prospective cohort study. Lancet Infect. Dis..

[46] Kayode A.J., Igbinosa E.O., Okoh A.I. (2020). Overview of listeriosis in the Southern African Hemisphere—Review. J. Food Saf..

[47] Opperman C.J., Bamford C. (2018). Co-infection with *Streptococcus pneumoniae* and *Listeria monocytogenes* in an immunocompromised patient. S. Afr. Med. J..

[48] Lalloo U.G., Coovadia Y.M., Adhikari M., Poyiadji O. (1992). *Listeria monocytogenes* meningitis at King Edward VIII Hospital, Durban. A 10-year experience, 1981–1990. S. Afr. Med. J..

[49] CLSI (2017). Performance Standards for Antimicrobial Susceptibility Testing.

[50] De Gans J., Van De Beek D. (2002). Dexamethason gunstig bij volwassenen met acute bacteriële meningitis; een gerandomiseerd placebogecontroleerd onderzoek. Ned. Tijdschr. Geneeskd..

[51] Temple M.E., Nahata M.C. (2000). Treatment of listeriosis. Ann. Pharmacother..

[52] Krawczyk-Balska A., Popowska M., Markiewicz Z. (2012). Re-evaluation of the significance of penicillin binding protein 3 in the susceptibility of *Listeria monocytogenes* to β-lactam antibiotics. BMC Microbiol..

[53] Van de Beek D., Cabellos C., Dzupova O., Esposito S., Klein M., Kloek A.T., Leib S.L., Mourvillier B., Ostergaard C., Pagliano P. (2016). ESCMID guideline: Diagnosis and treatment of acute bacterial meningitis. Clin. Microbiol. Infect..

[54] Hof H. (2004). An update on the medical management of listeriosis. Expert Opin. Pharmacother..

[55] Minkowski P., Staege H., Groscurth P., Schaffner A. (2001). Effects of trimethoprim and co-trimoxazole on the morphology of *Listeria monocytogenes* in culture medium and after phagocytosis. J. Antimicrob. Chemother..

[56] Michelet C., Leib S.L., Bentue-Ferrer D., Täuber M.G. (1999). Comparative efficacies of antibiotics in a rat model of meningoencephalitis due to *Listeria monocytogenes*. Antimicrob. Agents Chemother..

[57] Van de Velde S., Nguyen H.A., Van Bambeke F., Tulkens P.M., Grellet J., Dubois V., Quentin C., Saux M.-C. (2008). Contrasting effects of human THP-1 cell differentiation on levofloxacin and moxifloxacin intracellular accumulation and activity against *Staphylococcus aureus* and *Listeria monocytogenes*. J. Antimicrob. Chemother..

[58] Pagliano P., Brouwer M.C. (2016). Comment: “Implementation of a Meningitis Care Bundle in the Emergency Room Reduces Mortality Associated With Acute Bacterial Meningitis”. Ann. Pharmacother..

[59] Viale P., Scudeller L., Pea F., Tedeschi S., Lewis R., Bartoletti M., Sbrojavacca R., Cristini F., Tumietto F., Di Lauria N. (2015). Implementation of a Meningitis Care Bundle in the Emergency Room Reduces Mortality Associated with Acute Bacterial Meningitis. Ann. Pharmacother..

[60] Callapina M., Kretschmar M., Dietz A., Mosbach C., Hof H., Nichterlein T. (2001). Systemic and intracerebral infections of mice with *Listeria monocytogenes* successfully treated with linezolid. J. Chemother..

[61] Leiti O., Gross J.W., Tuazon C.U. (2005). Treatment of brain abscess caused by *Listeria monocytogenes* in a patient with allergy to penicillin and trimethoprim-sulfamethoxazole. Clin. Infect. Dis..

[62] Carryn S., Van Bambeke F., Mingeot-Leclercq M.-P., Tulkens P.M. (2003). Activity of β-lactams (ampicillin, meropenem), gentamicin, azithromycin and moxifloxacin against intracellular *Listeria monocytogenes* in a 24 h THP-1 human macrophage model. J. Antimicrob. Chemother..

[63] Stepanović S., Lazarević G., Ješić M., Koš R. (2004). Meropenem therapy failure in *Listeria monocytogenes* infection. Eur. J. Clin. Microbiol. Infect. Dis..

[64] Thønnings S., Knudsen J.D., Schønheyder H.C., Søgaard M., Arpi M., Gradel K.O., Østergaard C., Jensen U.S., Koch K., Smit J. (2016). Antibiotic treatment and mortality in patients with *Listeria monocytogenes* meningitis or bacteraemia. Clin. Microbiol. Infect..

[65] Chenal-Francisque V., Charlier C., Mehvish S., Dieye H., Leclercq A., Courvalin P., Lecuit M. (2014). Highly rifampin-resistant *Listeria monocytogenes* isolated from a patient with prosthetic bone infection. Antimicrob. Agents Chemother..

[66] Arsene O., Linassier C., Quentin R., Legras A., Colombat P. (1996). Development of listeriosis during vancomycin therapy in a neutropenic patient. Scand. J. Infect. Dis..

