# Supplementary material for: Antimicrobial-Resistant Listeria monocytogenes in Ready-to-Eat Foods: Implications for Food Safety and Risk Assessment
Source: Foods. 2023 Mar 22;12(6):1346. doi: 10.3390/foods12061346 (PMC10048454; doi:10.3390/foods12061346)
Supplement: Supplementary file 1 [file foods-12-01346-s001.zip › foods-2238921-Supplementary.pdf]

Table S1a. Antibiotic breakpoints for the description of the antibiotic susceptibility testing for *L. monocytogenes*

Antibiotic class	Antibiotic agents	Discs' code	Conc (µg)	Zones of inhibitions		
				Susceptible	Intermediate	Resistant
**β-Lactams**	Penicillin G	(P)	10	13	11-12	13
	Ampicillin	(AMP)	10	16	12-15	16
	Ampicillin-sulbactam	SAM	20	16	12-15	16
	Amoxicillin	(AML)	10	20	15-18	19
**Aminoglycosides**	Gentamicin	(CN)	10	17	15-16	12
	Amikacin	(AK)	30	17	15-16	14
	Streptomycin	(S)	25	17	15-16	14
**Carbapenems**	Doripenem	DOR	10	26	18-25	26
	Ertapenem	(ETP)	10	26	18-25	26
	Imipenem	(IPM)	10	26	18-25	26
**Cephalosporin**	Ceftriaxone	(CRO)	30	28	26-27	21
	Cefotetan	(CTT)	30	27	25-26	24
**Glycopeptides**	Vancomycin	(VA)	30	17	15-16	14
**Macrolides**	Erythromycin	(E)	15	21	16-20	15
	Clarithromycin	CLA	15	21	17-20	16
**Fluoroquinolones**	Ciprofloxacin	(CIP)	5	21	16-20	15
**Sulfonamides**	Trimethoprim	(W)	5	16	11-15	10
	Sulfamethoxazole	(RL)	5	19	16-18	15
	Trimethoprim-Sulfamethoxazole	TS	25 1.25/23.75	19	16-18	15
**Tetracyclines**	Oxytetracycline	(OT)	30	28	25-27	24
**Phenicol**	Chloramphenicol	(C)	30	21	18-20	20
**Phosphonic acid derivative**	Fosfomycin	(FOS)	50	16	13-15	12
**Colistin sulphate**		CO	10			

Antibiotic breakpoints were determined according to the Clinical and Laboratory Standards Institute and European Committee on Antimicrobial Susceptibility Testing (EUCAST) adopting the criteria set *L. monocytogenes, Streptococcus pneumonia,* and *Staphylococcus* spp (*14*, *22*).

Table S1b. Description of antibiotic susceptibility profile of *L. monocytogenes* isolates (n = 194) recovered from RTE food

Antibiotic class	Antibiotic agents	Discs′ code	Conc (μg)	No of Susceptible isolates (%)	Intermediate (%)	No of Resistant isolates (%)
**Prescribed antibiotics (PAs)**						
**β-Lactams**	Penicillin G	(P)	10	114 (58.8)	0	80 (41.24)
	Ampicillin	(AMP)	10	181 (93.3)	0	13 (6.70)
	Ampicillin-sulbactam	(SAM)	20	184 (94.9)	0	10 (5.16)
	Amoxicillin	(AML)	10	112 (57.7)	0	82 (42.27)
**Carbapenems**	Doripenem	(DOR)	10	162 (83.5)	0	32 (16.50)
	Ertapenem	(ETP)	10	123 (63.4)	0	71 (36.60)
	Imipenem	(IPM)	10	168 (86.6)	0	26 (13.40)
**Macrolides**	Erythromycin	(E)	15	53 (27.3)	63 (32.47)	78 (40.21)
	Clarithromycin	(CLA)	15	123 (63.40)	44 (22.68)	27 (13.92)
**Sulfonamides**	Trimethoprim	(W)	5	85 (43.81)	0	109 (56.19)
	Sulfamethoxazole	(RL)	5	66 (34.02)	8 (4.12)	120 (61.86)
	Trimethoprim-Sulfamethoxazole	(TS)	1.25/23.75	162 (83.51)	4 (2.06)	28 (14.43)
**Non-Prescribed antibiotics (non-PAs)**						
**Aminoglycosides**	Gentamicin	(CN)	10	143 (73.71)	33 (17.01)	18 (9.28)
	Amikacin	(AK)	30	146 (75.26)	13 (6.70)	35 (18.04)
	Streptomycin	(S)	25	87 (44.85)	29 (14.95)	78 (40.21)
**Cephalosporin**	Ceftriaxone	(CRO)	30	58 (29.90)	33 (17.01)	103 (53.09)
	Cefotetan	(CTT)	30	61 (31.44)	18 (9.28)	115 (59.28)
**Glycopeptides**	Vancomycin	(VA)	30	115 (59.28)	4 (2.06)	75 (38.66)
**Fluoroquinolones**	Ciprofloxacin	(CIP)	5	140 (72.17)	43 (22.17)	11 (5.67)
**Tetracyclines**	Oxytetracycline	(OT)	30	57 (29.38)	15 (7.73)	122 (62.89)
**Phenicol**	Chloramphenicol	(C)	30	150 (77.32)	19 (9.79)	25 (12.89)
**Phosphonic acid derivative**	Fosfomycin	(FOS)	50	159 (81.96)	20 (10.31)	15 (7.73)

Table S2. Multiple/Antibiotic resistance index of *L. monocytogenes* to EUCAST recommended and non-recommended antibiotics

Prescribed antibiotics (PAs)				Non-Prescribed antibiotics (non-PAs)		
Isolates no	MARPs	No of antibiotics	MARI	MARPs	No of antibiotics	MARI
Pol 1	ETP	1	0.08	CRO/CCT/OT	3	0.30
Pol 2	ETP/RL	2	0.17	CRO/CCT/VA/OT	4	0.40
Pol 3	ETP	1	0.08	CRO/CCT	2	0.20
Pol 4	E/RL	2	0.17	CRO/CCT/OT	3	0.30
Pol 5	W/RL	2	0.17	CCT	1	0.10
Pol 6	W/RL	2	0.17	CN/CCT/OT/FOS	4	0.40
Pol 7	-	-	-	S/OT	2	0.20
Pol 8	-	-	-	-	-	-
Pol 9	-	-	-	CCT	1	0.10
Pol 10	-	-	-	CCT/FOS	2	0.20
Pol 11	-	-	-	CCT/OT30	00.0.02	0.20
Pol 12	-	-	-	CCT/OT	2	0.20
Pol 13	-	-	-	CRO	1	0.10
Pol 14	AML/W/RL	2	0.17	S/CRO/VA/OT/FOS	5	0.50
Pol 15	AML/ETP/E/W/RL	5	0.17	CRO/VA/CIP/OT/C	5	0.50
Pol 16	E/RL	2	0.17	AK/S/CRO/CCT/OT	5	0.50
Pol 17	W/RL	2	0.17	AK/CRO/CCT/OT	4	0.40
Pol 18	AML/ETP/W/RL	4	0.33	S/VA/OT	3	0.30
Pol 19	AML/ETP/W/RL	4	0.33	S/VA	2	0.20
Pol 20	AML/DOR/CLA/W/RL/TS	6	0.5	S/CCT/VA	3	0.30
ARI	35 / 12 × 20 = 0.15	35		54 / 10 × 20 = 0.27	54	
Spol 1	P/AMP/SAM/AML/IPM/ETP/E/CLA/W/RL/TS	11	0.92	S/VA/CIP/OT/C	5	0.50
Spol 2	P/AMP/SAM/AML/IPM/E/CLA/W/RL/TS	10	0.83	S/VA/OT/C	4	0.40
Spol 3	P/AMP/SAM/IPM/E/CLA/W/RL/TS	9	0.75	CRO/VA/OT	3	0.30
Spol 4	P/AMP/SAM/IPM/E/CLA/W/RL/TS	9	0.75	CN/VA/OT/FOS	4	0.40
Spol 5	P/AMP/SAM/AML/E/CLA/W/RL/TS	9	0.75	S/CRO/CCT/VA/OT/C	6	0.60
Spol 6	P/AMP/SAM/DOR/E/RL/TS	7	0.58	CN/CRO/CCT/VA/CIP/OT/C	7	0.70
Spol 7	P/AMP/SAM/AML/IPM/E/CLA/W/RL/TS	10	0.83	S/CCT/VA/CIP/OT/C	6	0.60
Spol 8	P/AML/IPM/E/W/RL	6	0.5	S/CRO/VA/OT	4	0.40
Spol 9	P/AML/IPM/E/W/RL	6	0.5	S/CRO/CCT/VA/OT	5	0.50
Spol 10	P/AML/IPM/ETP/E/W/RL	7	0.58	S/CCT/VA/OT	4	0.40
Spol 11	RL	1	0.08	CN/AK/S/CRO/CCT/OT	6	0.60
Spol 12	W	1	0.08	CRO/CCT	2	0.20
Spol 13	AMP/ETP/E/W/RL	5	0.42	CRO/OT	2	0.20
Spol 14	AMP/ETP/TS	3	0.25	CCT/FOS	2	0.20
Spol 15	IPM	1	0.08	AK/CRO/CCT/OT	4	0.40
Spol 16	IPM	1	0.08	CRO/CCT/C	3	0.30
Spol 17	-	-	-	CRO/CCT	2	0.20
Spol 18	-	-	-	CCT/OT	2	0.20
Spol 19	-	-	-	CCT/OT	2	0.20
Spol 20	P/AML/W/RL	4	0.33	CN/S/VA	3	0.30
Spol 21	P/AML/W/RL	4	0.33	S/VA	2	0.20
Spol 22	P/AML/W/RL	4	0.33	S/CCT/VA	3	0.30
Spol 23	P/AML/W/RL	4	0.33	S/VA	2	0.20
ARI	112 / 12 × 23 = 0.41	112		83 / 10 × 23 = 0.36	83	
FS 1	DOR/IPM/ETP	3	0.25	CCT/OT	2	0.20
FS 2	IPM	1	0.08	CRO/CCT/OT	3	0.30
FS 3	IPM	1	0.08	CRO/CCT/CIP/OT	4	0.40
FS 4	-	-	-	CRO/CCT/OT	3	0.30

FS 5	W/RL	2	0.17	CN/S/CRO	3	0.30
FS 6	E/W/RL	3	0.25	CRO	1	0.10
FS 7	E/W/RL	3	0.25	CRO/CCT/OT	3	0.30
FS 8	-	-	-	OT	1	0.10
FS 9	AML	1	0.08	OT	1	0.10
FS 10	-	-	-	CRO/OT	2	0.20
FS 11	P/AML/DOR/IPM/E/W/RL	7	0.58	CN/AK/S/VA/OT/FOS	5	0.50
FS 12	IPM/W/RL/IPM	4	0.33	S/CRO/CCT	3	0.30
FS 13	P/AML/DOR/IPM/E/CLA/W/RL	8	0.66	CRO/VA/OT/C	4	0.40
FS 14	P/AML/E/W/RL	5	0.42	CRO/VA/OT/C	4	0.40
FS 15	P/AML/E/W/RL	5	0.42	S/CRO/VA/OT/C	5	0.50
FS 16	P/AML/E/W/RL	5	0.42	CRO/VA/OT/C	4	0.40
FS 17	DOR/RL	2	0.17	CCT/VA	2	0.20
FS 18	AML/RL	2	0.17	S/CRO/CCT/VA	4	0.40
FS 19	IPM/RL	2	0.17	CRO/CCT/VA	3	0.30
FS 20	AML/DOR/RL	3	0.25	CN/S/CRO/VA	4	0.40
FS 21	P/AML/DOR/E/W/RL	6	0.50	AK/S/OT	3	0.30
FS 22	AML/E/W/RL	4	0.33	AK/OT/C	3	0.30
FS 23	RL	1	0.08	AK/CRO/CCT/OT/C	5	0.50
FS 24	AML/IPM/RL	3	0.25	CN/AK/S/CRO/CCT/OT/C	7	0.70
FS 25	DOR/RL	2	0.17	CRO/CCT	2	0.20
FS 26	W/RL	2	0.17	AK/CCT	2	0.20
FS 27	P/AML/DOR/E/W/RL	6	0.50	CCT/VA	2	0.20
FS 28	P/AML/DOR/IPM/ETP/E/CLA/W/RL	9	0.75	CRO/CCT/VA/OT	4	0.40
FS 29	AML/IPM/RL	3	0.25	S/CRO/CCT/OT	4	0.40
FS 30	AML/IPM	2	0.17	AK/S/CRO/CCT/OT	5	0.50
ARI	95 / 12 × 30 = 0.26	95		98 / 10 × 30 = 0.33	98	
Ch 1	IPM/ETP	2	0.17	CCT/OT	2	0.20
Ch 2	RL	1	0.08	CCT/OT/C	3	0.30
Ch 3	ETP	1	0.08	CCT	1	0.10
Ch 4	IPM	1	0.08	CRO/CCT/OT	3	0.30
Ch 5	IPM	1	0.08	CRO/CCT/OT	3	0.30
Ch 6	IPM	1	0.08	CRO/CCT/OT	3	0.30
Ch 7	IPM/W	2	0.17	S/CCT/OT	3	0.30
Ch 8	ETP	1	0.08	CCT/OT/C	3	0.30
Ch 9	-	-	-	CRO/CCT/OT	3	0.30
Ch 10	P/AML	2	0.17	S/CCT/VA/OT/C	5	0.50
Ch 11	AML/E/CLA	3	0.25	CCT	1	0.10
Ch 12	-	-	-	CCT/FOS	2	0.20
Ch 13	ETP/W	2	0.17	CCT	1	0.10
Ch 14	-	-	-	CCT	1	0.10
Ch 15	-	-	-	CCT	1	0.10
Ch 16	P/AMP/SAM/AML/DOR/CLA/W/RL/TS	9	0.75	S/CRO/CCT/VA/OT	5	0.50
ARI	26 / 12 × 16 = 0.14	26		40 / 10 × 16 = 0.25	40	
FF 1	W	1	0.08	CRO/CCT/OT	3	0.30
FF 2	IPM	1	0.08	CRO/CCT/OT	3	0.30
FF 3	IPM	1	0.08	CRO/CCT/OT	3	0.30
FF 4	-	-	-	CCT/OT	2	0.20
FF 5	IPM/ETP/E	3	0.25	CRO/CCT/OT	3	0.30
FF 6	IPM/ETP	2	0.17	CRO/CCT/OT	3	0.30
FF 7	ETP	1	0.08	CRO/CCT/OT	3	0.30
FF 8	P/AML/IMP/E/W/RL	6	0.50	CN/S/CCT/VA/OT	5	0.50
FF 9	P/AML/IPM/W	4	0.33	CRO/CCT/OT	3	0.30
FF 10	P/AML/IPM/W	4	0.33	CCT/VA/OT/C	4	0.40
FF 11	P/AML/IPM/W	4	0.33	CCT/VA/OT/C	4	0.40

FF 12	P/AML/W/RL	4	0.33	S/VA/OT	3	0.30
FF 13	P/AML/DOR/W/RL	5	0.42	AK/CRO/CCT	3	0.30
FF 14	P/AML/W/RL	4	0.33	S/CRO/CCT/VA	4	0.40
FF 15	P/AML/W/RL	4	0.33	AK/S/CRO/CCT/VA	5	0.50
FF 16	P/AML/W/RL	4	0.33	AK/S/CRO/CCT/VA	5	0.50
FF 17	AML/ETP/RL	3	0.25	AK/S/CRO/CCT	4	0.40
FF 18	P/AML/DOR/CLA/W/RL/TS	7	0.58	AK/S/CRO/CCT/VA/OT	6	0.60
FF 19	P/AML/DOR/CLA/W/RL/TS	7	0.58	AK/S/CRO/CCT/VA	5	0.50
FF 20	P/AML/DOR/CLA/W/RL/TS	7	0.58	AK/S/CRO/CCT	4	0.40
FF 21	AML/DOR/CLA/W/RL/TS	6	0.50	CN/AK/S/CRO/CCT/VA	6	0.60
ARI	78 / 12 × 21 = 0.31	78		81 / 10 × 21 = 0.39	81	
RS 1	P/AML/E/RL	4	0.33	CRO/CIP/OT	3	0.30
RS 2	AML/W/RL	3	0.25	CRO/OT	2	0.20
RS 3	IPM	1	0.08	CRO/CCT/OT	3	0.30
RS 4	AML/IPM	2	0.17	CRO	1	0.10
RS 5	P/AML/E/RL	4	0.33	CN/S/CCT/OT	4	0.40
RS 6	IPM/RL	2	0.17	CCT/OT	2	0.20
RS 7	-	-	-	S/OT/FOS	3	0.30
RS 8	AML/E/W/RL	4	0.33	S/OT/FOS	3	0.30
RS 9	AML/W/RL	3	0.25	CIP/OT/C	3	0.30
RS 10	E/W/RL	3	0.25	S/FOS	2	0.20
RS 11	AML/RL/TS	3	0.25	S/CRO/CCT/OT/C	5	0.50
RS 12	AML/E/W/RL/TS	5	0.42	S/CCT	2	0.20
RS 13	P/AML/W/RL	4	0.33	CCT/VA	2	0.20
RS 14	AMP/SAM/AML/DOR/CLA/W/RL/TS	8	0.67	S/CCT/VA	3	0.30
ARI	46 / 12 × 14 = 0.27	46		48 / 10 × 14 = 0.34	38	
RV 1	P/AML/IMP/W/RL	5	0.42	VA	1	0.10
RV 2	AML/IPM	2	0.17	VA/OT	2	0.20
RV 3	AMP/SAM/AML/IPM/W/RL/TS	7	0.58	VA/OT	2	0.20
RV 4	P/AML/W/RL	4	0.33	S/VA/OT	3	0.30
ARI	18 / 12 × 4 = 0.38	18		8 / 10 × 4 = 0.2	8	
Bd 1	IPM/E/W	3	0.25	AK/S/CRO/CTT/OT	5	0.50
Bd 2	P/AML/E/W/RL	5	0.42	VA/OT/C	3	0.30
Bd 3	P/AML/DOR/E/CLA/W/RL/TS	8	0.67	S/VA/OT	3	0.30
Bd 4	E/W/RL	3	0.25	CN/S/CRO/CTT/VA	5	0.50
Bd 5	IPM/E/W/RL	4	0.33	CN/CRO/CTT/VA/OT	5	0.50
Bd 6	P/AML/DOR/IPM/E/W/RL	7	0.58	CN/S/CRO/CTT/VA	5	0.50
Bd 7	DOR/E/W/RL	4	0.33	AK/S/CRO/CTT/OT	5	0.50
Bd 8	P/W/RL	3	0.25	VA	1	0.10
Bd 9	AML/DOR/E/CLA/W/RL/TS	7	0.58	AK/S/CRO/CTT/VA	5	0.50
Bd 10	P/AML/E/CLA/W/RL	6	0.50	S/CRO/CTT/VA	4	0.40
Bd 11	P/AML/E/CLA/W/RL	6	0.50	AK/S/CRO/CTT/VA	5	0.50
ARI	56 / 12 × 11 = 0.42	56		46 / 10 × 11 = 0.42	46	
Fc 1	AML/RL	2	0.17	AK/CRO/CTT	3	0.30
Fc 2	IPM/E/W	3	0.25	CRO/CTT/CIP	3	0.30
ARI	5 / 12 × 2 = 0.21	5		6/10 × 2 = 0.3	6	
Ps 1	IPM/ETP/E/RL	4	0.33	AK/CRO/CCT/OT	4	0.40
Ps 2	P/AML/IPM/ETP/E/W	6	0.50	CRO/VA/CIP/OT/C	5	0.50
Ps 3	IPM/ETP/W/RL	4	0.33	CCT/VA/CIP/OT	4	0.40
Ps 4	P/IPM/ETP/E/W/RL	6	0.50	CCT/VA/OT	3	0.30
Ps 5	AML/DOR/IPM	3	0.25	AK/CRO/CCT/OT	4	0.40
Ps 6	P/AML/IPM/E/W/RL	6	0.50	CN/CCT/VA/OT	4	0.40
Ps 7	AML/IPM/W	3	0.25	CRO/CCT/OT	3	0.30
Ps 8	-	-	-	CCT	1	0.10
Ps 9	P/AML/IPM/E/W/RL	6	0.50	CCT/VA/OT	3	0.30

Ps 10	-	-	-	CCT	1	0.10
Ps 11	IPM	1	0.08	CCT	1	0.10
Ps 12	AML/IPM/E/W/RL	5	0.42	CRO/CCT/OT/C	4	0.40
Ps 13	-	-	-	CRO/OT	2	0.20
Ps 14	AML	1	0.08	CRO	1	0.20
Ps 15	-	-	-	-	-	-
Ps 16	P/IPM/W/RL	4	0.33	AK/CRO/CCT/VA/OT	5	0.50
Ps 17	E/W/RL/TS	4	0.33	AK/CRO/CCT/OT	4	0.40
Ps 18	E/CLA/W/RL/TS	5	0.42	AK/CRO/CCT/OT	4	0.40
Ps 19	P/AML/DOR/CLA/W/RL/TS	7	0.58	AK/CRO/CCT/VA/OT/C	6	0.60
Ps 20	P/AML/E/CLA/W/RL/TS	7	0.58	AK/CRO/CCT/VA/OT	5	0.50
Ps 21	P/AML/CLA/W/RL/TS	6	0.50	AK/CRO/CCT/VA/OT	5	0.50
Ps 22	P/AMP/CLA/W/RL/TS	6	0.50	CRO/CCT	2	0.20
ARI	84 / 12 × 22 = 0.32	84		71/10 × 22 = 0.32	71	
Cc 1	W	1	0.08	CRO/CCT/OT/C	4	0.40
Cc 2	AML	1	0.08	CCT/OT	2	0.20
Cc 3	P/DOR/E/W/RL	5	0.42	CRO/CCT/OT/FOS	4	0.40
Cc 4	P/E/W/RL	4	0.33	OT	1	0.10
Cc 5	P/AML/IPM/W/RL	5	0.42	S/VA	2	0.20
Cc 6	P/AML/DOR/IPM/W/RL	6	0.50	S/VA	2	0.20
Cc 7	P/IPM/E/W/RL	5	0.42	AK/S/CRO/CCT/VA/OT	6	0.60
Cc 8	P/DOR/IPM/E/W/RL	6	0.50	S/CCT/OT	3	0.30
Cc 9	P/AML/IPM/W/RL	5	0.50	S/CRO/CCT/VA/CIP	5	0.50
Cc 10	DOR/ETP/E/CLA/W/RL/TS	7	0.58	S/CRO/CCT/VA/OT	5	0.50
ARI	45 / 12 × 10 = 0.38	45		34 / 10 × 10 = 0.34	34	
Mu 1	IPM/RL	2	0.17	AK/S/CRO/CCT/OT	5	0.50
Mu 2	DOR	1	0.08	S/CRO/CCT/OT	4	0.40
Mu 3	E/CLA/W	3	0.25	CCT/OT	2	0.20
Mu 4	-	-	-	CCT/OT	2	0.20
Mu 5	P/E/W/RL	4	0.33	CCT/OT	2	0.20
Mu 6	-	-	-	OT	1	0.10
Mu 7	-	-	-	CCT/OT	2	0.20
Mu 8	RL	1	0.08	OT	1	0.10
Mu 9	RL	1	0.08	CRO/CCT/VA/OT	4	0.40
Mu 10	P/AML/W/RL	4	0.33	S/CCT/FOS	3	0.30
Mu 11	P/AML/W/RL	4	0.33	CCT/VA/FOS	3	0.30
Mu 12	P/AML/DOR/CLA/W/RL/TS	7	0.58	CCT/VA/FOS	3	0.30
ARI	27 / 12 × 12 = 0.19	27		32 / 10 × 12 = 0.27	32	
AS 1	IPM	1	0.08	CRO/CCT/OT	3	0.30
AS 2	IPM	1	0.08	CRO/CCT/OT	3	0.30
AS 3	IPM	1	0.08	CRO/CCT/OT	3	0.30
AS 4	AML/IPM/E/W/RL	5	0.42	CN/S/CCT/OT/FOS	5	0.50
AS 5	IPM	1	0.08	CCT	1	0.10
AS 6	P/E/W/RL	4	0.33	CCT	1	0.10
AS 7	IPM	1	0.08	S/CCT	2	0.20
AS 8	-	-	-	CCT	1	0.10
AS 9	P	1	0.08	CRO/CCT	2	0.20
ARI	15 / 12 × 9 = 0.14	15		21 / 10 × 9 = 0.23	21	

Pol - polony, Spol - sliced polony, FS - fruit salad, Ch – potato chips, FF - fried fish, RS - Russian sausage, RV - red Vienna, Bd – bread, FC - fried chicken, Vk - vetkoek, Ps - pie, Cc - cupcakes, Mu - muffins, AS - assorted sandwiches.

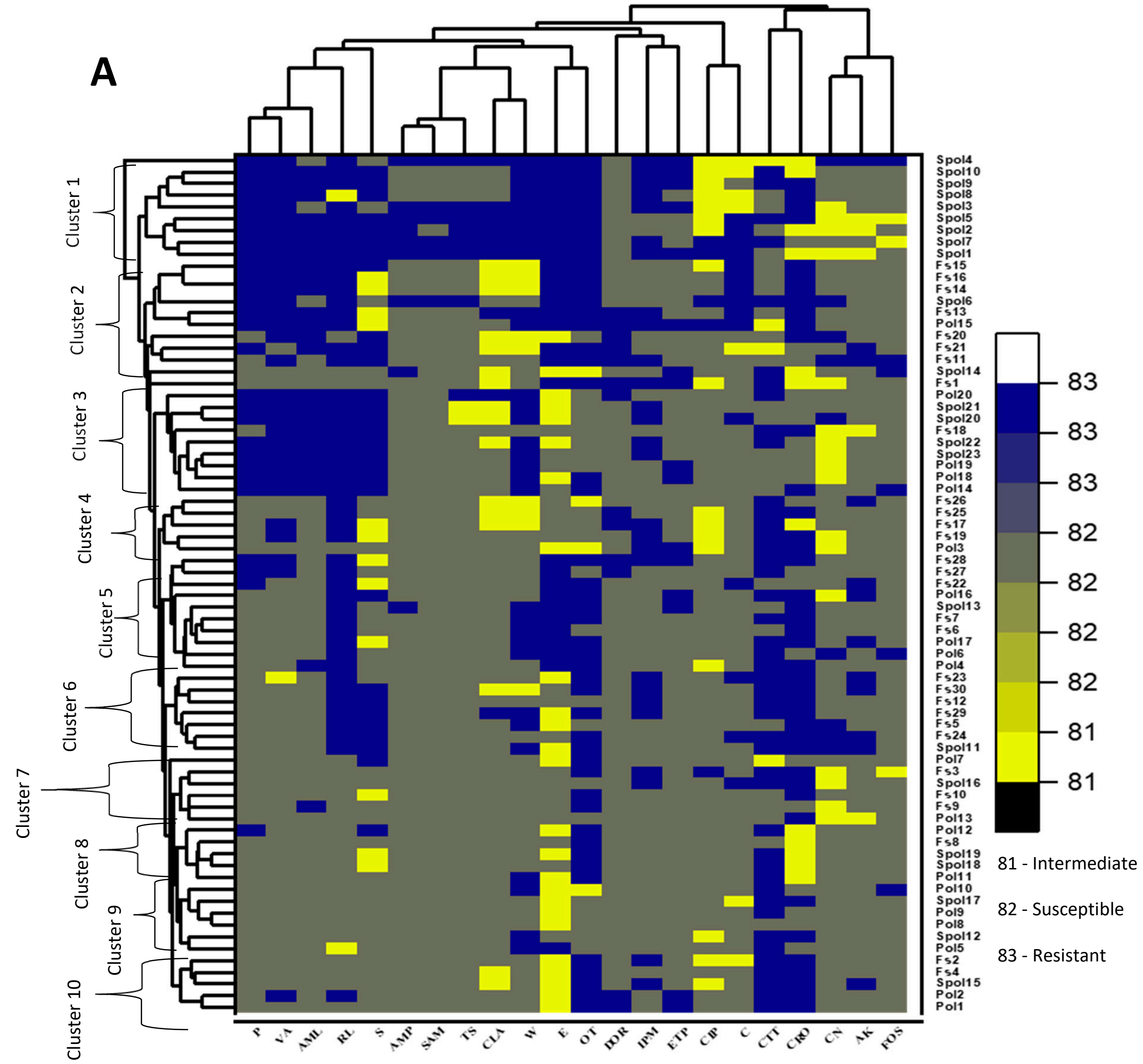

Figure S1. Heatmap cluster analysis of *L. monocytogenes* isolates from ready-to-eat foods. The Column and row clusters grouped isolates and antimicrobials according to the response/susceptibility.

The food samples (y-axis) are denoted by the acronyms (Pol - polony, Spol - sliced polony, FS - fruit salad, Ch – potato chips, FF - fried fish, RS - Russian sausage, RV - red Vienna, Bd – bread, FC - fried chicken, Vk - vetkoek, Ps - pie, Cc - cupcakes, Mu - muffins, AS - assorted sandwiches) and the respective isolates number from the samples corresponds with the column "isolate no" in Table S4 (i.e. Spol 4 is interpreted as sliced polony isolate number "4").

Antimicrobials: Penicillin G (P), Ampicillin (AMP), Ampicillin-sulbactam (SAM), Amoxicillin (AML), Gentamicin (CN), Amikacin (AK), Streptomycin (S), Doripenem (DOR), Ertapenem (ETP), Imipenem (IPM), Ceftriaxone (CRO), Cefotetan (CTT), Vancomycin (VA), Erythromycin (E), Clarithromycin CLA, Ciprofloxacin (CIP), Trimethoprim (W), Sulfamethoxazole (RL), Trimethoprim-Sulfamethoxazole (TS), Oxytetacyclin (OT), Chloramphenicol (C), Fosfomycin (FOS) are represented on the x-axis.

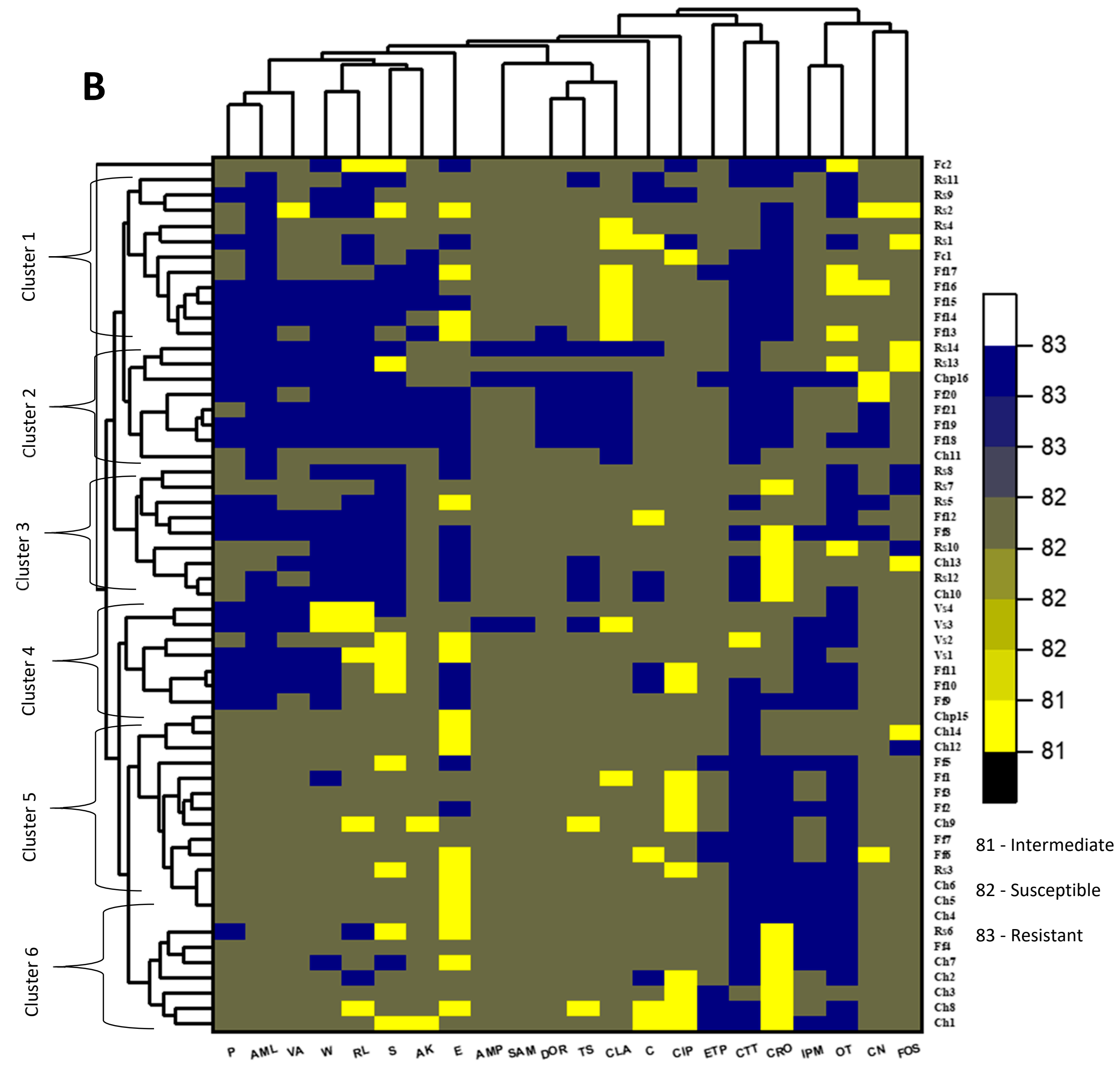

Figure S2. Heatmap cluster analysis of *L. monocytogenes* isolates from ready-to-eat foods. The Column and row clusters grouped isolates and antimicrobials according to the response/susceptibility.

The food samples (y-axis) are denoted by the acronyms (Pol - polony, Spol - sliced polony, FS - fruit salad, Ch – potato chips, FF - fried fish, RS - Russian sausage, RV - red Vienna, Bd – bread, FC - fried chicken, Vk - vetkoek, Ps - pie, Cc - cupcakes, Mu - muffins, AS - assorted sandwiches) and the respective isolates number from the samples corresponds with the column “isolate no” in Table S4 (i.e. Spol 4 is interpreted as sliced polony isolate number “4”).

Antimicrobials: Penicillin G (P), Ampicillin (AMP), Ampicillin-sulbactam (SAM), Amoxicillin (AML), Gentamicin (CN), Amikacin (AK), Streptomycin (S), Doripenem (DOR), Ertapenem (ETP), Imipenem (IPM), Ceftriaxone (CRO), Cefotetan (CTT), Vancomycin (VA), Erythromycin (E), Clarithromycin CLA, Ciprofloxacin (CIP), Trimethoprim (W), Sulfamethoxazole (RL), Trimethoprim-Sulfamethoxazole (TS), Oxytetacyclin (OT), Chloramphenicol (C), Fosfomycin (FOS) are represented on the x-axis.

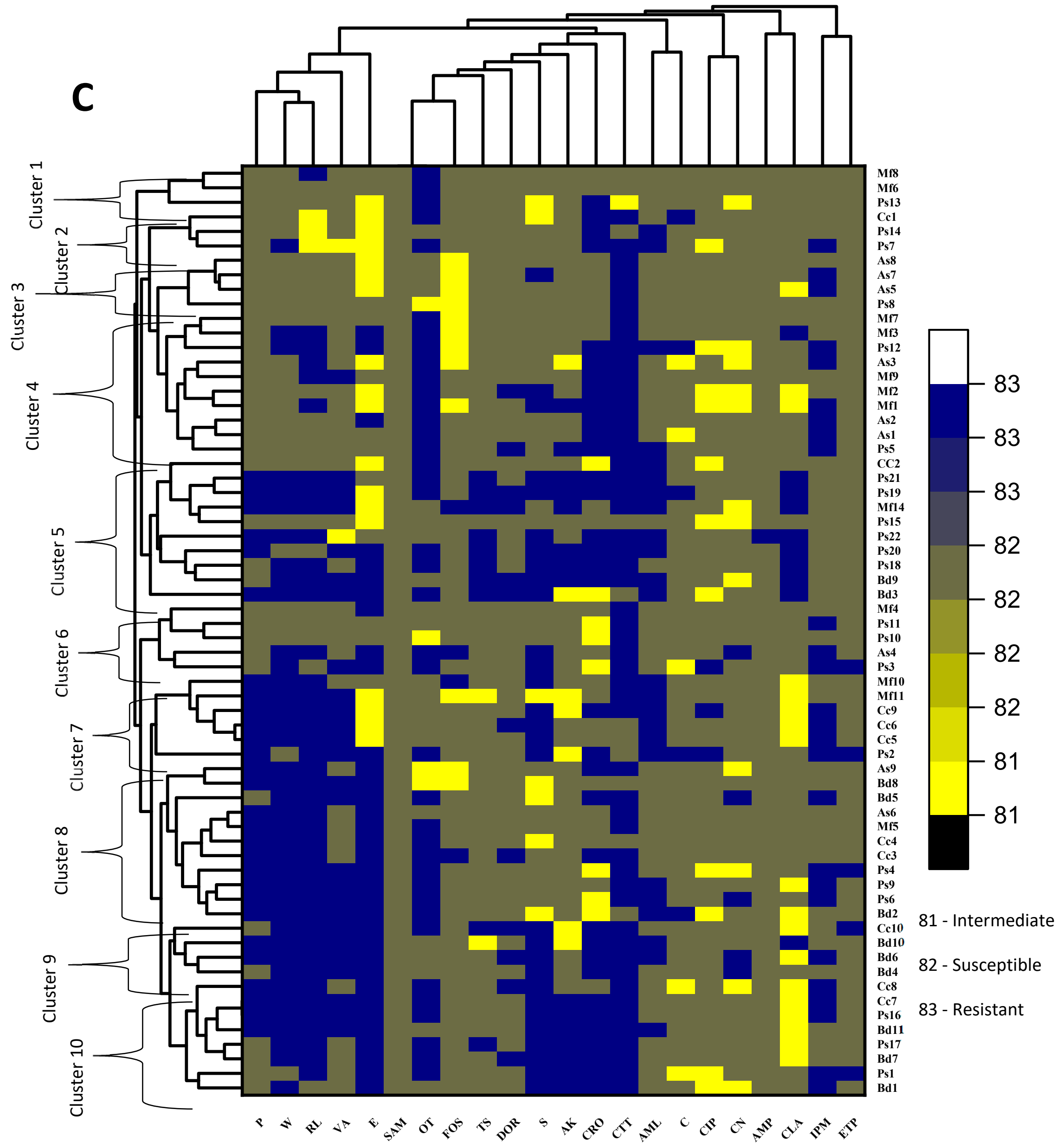

Figure S3. Heatmap cluster analysis of *L. monocytogenes* isolates from ready-to-eat foods. The Column and row clusters grouped isolates and antimicrobials according to the response/susceptibility.

The food samples (y-axis) are denoted by the acronyms (Pol - polony, Spol - sliced polony, FS - fruit salad, Ch – potato chips, FF - fried fish, RS - Russian sausage, RV - red Vienna, Bd – bread, FC - fried chicken, Vk - vetkoek, Ps - pie, Cc - cupcakes, Mu - muffins, AS - assorted sandwiches) and the respective isolates number from the samples corresponds with the column "isolate no" in Table S4 (i.e. Spol 4 is interpreted as sliced polony isolate number "4").

Antimicrobials: Penicillin G (P), Ampicillin (AMP), Ampicillin-sulbactam (SAM), Amoxicillin (AML), Gentamicin (CN), Amikacin (AK), Streptomycin (S), Doripenem (DOR), Ertapenem (ETP), Imipenem (IPM), Ceftriaxone (CRO),

Cefotetan (CTT), Vancomycin (VA), Erythromycin (E), Clarithromycin CLA, Ciprofloxacin (CIP), Trimethoprim (W), Sulfamethoxazole (RL), Trimethoprim-Sulfamethoxazole (TS), Oxytetacyclin (OT), Chloramphenicol (C), Fosfomycin (FOS) are represented on the x-axis.
